# Development of hamming and hausdorff distance metrics for cubic intuitionistic fuzzy hypersoft set in cement storage quality control: Development and evaluation

**DOI:** 10.1371/journal.pone.0291817

**Published:** 2023-09-25

**Authors:** Muhammad Saeed, Muhammad Haris Saeed, Misbah Khalid, Ibrahim Mekawy

**Affiliations:** 1 Department of Mathematics, University of Management and Technology, Lahore, Pakistan; 2 Department of Chemistry, University of Management and Technology, Lahore, Pakistan; 3 Department of Mathematics, College of Science and Arts, Qassim University, Al-Rass, Kingdom of Saudi Arabia; Korea National University of Transportation, KOREA, DEMOCRATIC PEOPLE’S REPUBLIC OF

## Abstract

Quality control is paramount in product manufacturing as it ensures consistent production to meet customer expectations, regulatory requirements and maintain a company’s reputation and profitability. Distance measures within fuzzy sets serve as powerful tools for quality control, allowing for data comparison and identification of potential defects or outliers within a system. This study aims to develop a hybrid concept by combining a Cubic Intuitionistic Fuzzy Set (CIFS) with Soft Set (SS) and extending it to Cubic Intuitionistic Fuzzy Hypersoft Set (CIFHSS). CIFHSS enables handling multiple distinct attributes at the sub-attribute level within a cubic set environment. The concept includes operations like internal, partial internal, external, complement, direct sum, and product. Additionally, six distance metrics are defined within CIFHSS and applied to establish a quality control management system for industrial applications. The versatility of CIFHSS in quality control management stems from its ability to capture and model uncertainty, vagueness, and imprecision in data. This makes it an effective tool for decision-making, risk analysis, and process optimization across a wide range of industrial applications.

## 1 Introduction

In order to ensure that the goods satisfy the necessary quality, safety, and reliability criteria, quality control is a vital component of manufacturing. Traditional quality control procedures rely on precise, well-defined data and statistical analysis to identify deviations and anomalies. However, adopting traditional quality control procedures can be difficult in many industrial applications since the data is frequently ambiguous, unclear, or lacking. When it comes to dealing with ambiguous and uncertain data in quality control management, fuzzy logic and fuzzy sets offer a potent option. Zadeh [1] put forward the idea of Fuzzy Sets (FS) where a value between [0, 1] is assigned to an element in a set. This mathematical framework extends classical logic by allowing partial truth values between true and false. This enables the representation of uncertain and vague information often encountered in real-world problems. Fuzzy sets can capture and represent the degree of uncertainty and vagueness inherent in real-world data, making them a powerful tool for modelling and decision-making.

Uncertainty can arise from a variety of places when discussing multi-attribute problems such as:

1. The decision maker (DMs) are not provided with accurate information on which is to be accessed to make decisions.
2. The DMs may not be well informed on the subject on which they are making the decisions.
3. The inability of the decision maker to explicitly discriminate how one alternative is superior over the other during the decision-making process [2].

In the third condition, the DMs access the alternatives using a membership degree instead of providing an assessment with complete certainty [3].

The concept of FS was then extended to interval-valued fuzzy sets (IVFS) by Zadeh in 1975 [4]. This extension allowed for the division of the membership function into an interval instead of a single value which is more suited to real-world applications. Atanassov [5] introduced an intuitionistic fuzzy set (IFS) in 1986, taking into account not only the degree of membership of an element to a set but also the degree of non-membership and the degree of hesitation of the decision-maker in assigning a membership grade. Numerous researchers have extensively explored the concept of similarity measures (SM) within the domain of Intuitionistic Fuzzy Sets (IFSs). The journey began with Atanassov (1993), who initially defined SM for IFS elements [6]. Building upon this foundation, Chen (1995) introduced the idea of quantifying similarity degrees for vague sets [7]. However, Hong and Kim (1999) noted that Chen’s measures exhibited inaccuracies and indistinguishable outcomes under extreme conditions, necessitating the development of modified SM [8]. Subsequently, Dengfeng and Chuntian (2002) focused on determining SMs for IFSs, especially in the context of discrete or continuous universal sets for pattern recognition problems [9]. Nevertheless, Mitchell (2003) identified limitations in Dengfeng and Chuntian’s measures, as they also produced indistinguishable results in specific scenarios [10]. To overcome these challenges, Hung and Yang (2004) proposed SM based on the Hausdorff distance for IFSs, providing a foundation for deriving essential properties [11]. Expanding the scope, Szmidt and Kacprzyk (2004) introduced SMs to address medical diagnosis problems utilizing IFS [12]. Following suit, Liu (2005) modified Hong and Kim’s SM and explored the relevant properties thereof [13]. Additionally, Liang and Shi (2003) exchanged SMs between pairs of IFSs, comparing their performance against existing ones through numerical examples [14]. Ye (2011) investigated cosine SM for IFS, uncovering its unique characteristics [15]. Furthermore, Wang and Xin (2005) delved into the relationship between different SM measures and their application in pattern recognition problems [16]. As research continued to progress, Song et al. (2014) introduced weighted SMs for IFSs, catering to scenarios where varying importance levels were assigned to elements [17]. Ngan et al. (2018) explored distance-based measures using H-max measures for IFS information, opening new avenues for analysis. Notably, Khan and Lohani (2016) contributed by discussing clustering algorithms based on distance measures, promoting effective data organization techniques [18, 19]. Numerous other scholars and researchers continue exploring the vast landscape of IFS similarity measures, each striving to address specific problems and uncover novel perspectives [20–23]. Similarly, highly complex and versatile structures have been developed in various industrial applications, including medical diagnostics, decision-making, pattern recognition, and risk analysis from these hybrid fuzzy structures [24–27].

In 2012, Jun et al. [28] combined the concept of IVFS and FS to develop a Cubic Set (CS) and developed properties like internal cubic sets (ICS) and external cubic sets (ECS). Cubic sets help us evaluate the same object’s range at a particular event. Cubic fuzzy sets are a powerful extension of fuzzy sets that provide a higher degree of flexibility and expressiveness in modeling uncertain data. In fuzzy cubic sets, the degree of membership is represented by a cubic function, which allows for a more precise and detailed representation of uncertainty. Within this context, Khan et al. [29] introduced specific cubic aggregation operators. In contrast, Mahmood et al. [30] explored the notion of cubic hesitant fuzzy sets and their corresponding aggregation operators for decision-making. However, these theories primarily focused on membership intervals and did not adequately address the crucial role of non-membership information in the decision-making process. Non-membership data entities play an equally significant role in the assessment of alternatives, warranting further attention in the research.

The concept of soft set was extended to Hypersoft set (HS) by Smarandache in 2018. Hypersoft set allowed for the division of attributes used in soft sets to be further divided into sub-attributes such that the selected attributes are disjoint from one another [31]. This division of attributes to sub-attributes opened a pathway for more approaching and processing information more concisely and detailed, leading to better results. This concept was further hybridized with many fuzzy structures to increase the ability to apply to different scenarios each addressing something different from the other. For example, a variety of neutrosophic and complex fuzzy hypersoft structures have been used in literature for developing disease diagnosis models [32–35]. A hybrid of picture fuzzy graph and hypersoft set was developed to address micro-enterprise investment risk assessment [36]. Also, a number of similar hypersoft set structures have been found in literature and applied in different decision-making scenarios [37–39].

The application of Cubic Intuitionistic Fuzzy Sets (CIFS) has yielded a range of fuzzy hybrid structures, documented across diverse literature for various applications.

The application of Cubic Intuitionistic Fuzzy Sets (CIFS) has yielded a range of fuzzy hybrid structures, documented across diverse literature for various applications. Mahmood et al. (2016) extended this concept to introduce Cubic Hesitant Fuzzy Sets (CHFS) as an efficient tool for decision-making processes regarding performance of imaging techniques for diagnosis of breast cancer [40]. Furthermore, Kaur and Garg (2018b) contributed by presenting generalized aggregation operators (AOs) tailored for Cubic Intuitionistic Fuzzy Numbers (CIFNs), offering innovative solutions to decision-making problems [41]. In a similar vein, Garg and Kaur (2018) devised a TOPSIS method grounded in distance measures within the CIFS framework, facilitating the resolution of group decision-making challenges [42]. Senapati et al. (2015) took the concept of Cubic Sets to the next level, exploring subalgebras, ideals, and introducing closed ideals within the realm of B-algebras [43]. Additionally, Kang and Kim (2016) delved into the theory of images and inverse images of cubic sets [44]. Similarly, Saqlain et al. (2022) extended the concept of CIFS to cubic intuitionistic fuzzy soft set allowing for addressing individual attributes in a decision-making problem [45]. Notably, the existing CIFSS framework lacks consideration for the NMD corresponding to MD, limiting its applicability. Remarkably, there has been no prior exploration into the development of hybrid structures combining Hypersoft sets and CIFS, which promises to provide a deeper understanding of decision-making attributes by dividing each of the attributes into sub-attributes, paving the way for more effective and efficient decision-making processes. The development of the hybrid fuzzy structure called the Cubic Intuitionistic Fuzzy Hypersoft Set, was motivated by the need for a versatile analysis tool to comprehensively evaluate at a sub-attribute level. Traditional fuzzy set theory and its extensions have proven effective in handling uncertainty and vagueness in various applications. However, there was a need to enhance the existing frameworks to better represent complex decision-making scenarios, particularly in the context of industrial quality control. The CIFS is a powerful extension of fuzzy set theory that incorporates two membership functions (i.e., membership and non-membership) and two fuzzy intervals (i.e., membership and non-membership intervals) that either contain the membership and non-membership functions or don’t. This enables a more accurate representation of uncertainty and ambiguity in decision-making processes. On the other hand, Hypersoft set theory focuses on analyzing attribute-based data from soft set theory at a sub-attribute level. Combining these two frameworks, the CIFHS was developed to provide a comprehensive and flexible approach to decision analysis. The CIFHS structure allows for a granular analysis at a sub-attribute level, enabling a detailed examination of various factors influencing industrial quality control. It represents the inherent ambiguity and imprecision in quality control procedures, which are frequently influenced by a number of variables and judgment calls. The CIFHS framework offers a more accurate and complete depiction of the quality control environment by utilizing the CIFS and Hypersoft set components. Hamming and Hausdorff distance metrics were created to improve the CIFHS structure’s practical applicability even more. These distance measurements make it possible to quantitatively compare or contrast various quality control samples or sub-attributes. By comparing various data points, spotting abnormalities, and spotting patterns or trends that could affect product quality, the CIFHS framework uses these measures to support efficient quality control analysis. The CIFHS structure and its corresponding distance measures can be used in a variety of ways for industrial quality control. First, it may be used to model and analyze intricate quality control systems while taking into account a variety of sub-attributes and how they interact. This makes it possible for decision-makers to evaluate how various aspects affect overall quality and take appropriate action. Second, distance measurements enable quantitative evaluation of sample variability, assisting in the detection of outliers or departures from predetermined quality standards. Such anomalies can be quickly identified and the necessary remedial actions can be done to provide constant quality control. The major objective is to improve quality control in the production of industrial products by addressing uncertainty, ambiguity, and imprecision in data effectively. CIFHSS enables the management of multiple distinct attributes at the sub-attribute level within a cubic set environment, providing a more detailed analysis of complex industrial systems.

In this study, we generalize the CIFS to a cubic intuitionistic fuzzy soft set (CIFSS) and introduced a hybrid of CIFHS (CIFHSS). CIFHSS deals with multiple distinct attributes in the CS environment. We introduced the concept of Internal Cubic (resp. External Cubic) Intuitionistic Fuzzy Hypersoft set, and Internal Cubic (resp. External Cubic). Furthermore, complementing these sets and discussing some aggregation operators with the examples introduced distance measures of CIFHSS and a decision-making approach.

The study is organized in the following manner: Section 2 provides some selected preliminary definitions essential for introducing CIFHSS. Section 3 defines the concept of CIFHS with the operators mentioned above and properties. Section 4 focuses on first introducing the applications of fuzzy distance measures and then developing Hamming and Hausdorff distance measures along with proofs to validate their applications. Section 5 illustrates the application of the developed distance measures in industrial quality control by developing an algorithm for computation of similarity from an ideal solution. This concept is then applied to selecting ideal cement storage facilities out of a group of options by comparison of the facilities to a reference set. The paper is then rounded up with major findings and future works in the Conclusion section.

## 2 Preliminaries

This section provides some essential definitions that will be used further on in the article:

**Definition 1.1**. [45] *Suppose* ℧ *be a set of discourse, Ë be an attributive set with respect to* ℧ *and* P *the subset of Ë. Then, soft set will be*:

$$M_{P}=\left\{\left\langle \dot{e},M\left(\dot{e}\right)\right\rangle |\dot{e}\in P,M\left(\dot{e}\right)\in P^{℧}\right\}$$

*where M is a mapping such that*:

$$M:P\to P^{℧}$$

*and* *P*^℧^ *representing the collection of all subsets of* ℧. $\langle \dot{e},M\left(\dot{e}\right)\rangle$ *is an ordered pair where* $\dot{e}$ are the attributes from set P⊆Ë.

**Definition 1.2**. [46] *Suppose* ℧ *be a set of discourse. Ë be an attributive set with respect to* ℧ *and* P *the subset of Ë. Then Fuzzy Soft Set can be expressed as*:

$$M_{P}=\left\{\left\langle \dot{e},M\left(\dot{e}\right)\right\rangle |\dot{e}\in P,M\left(\dot{e}\right)\in F^{℧}\right\}$$

*where M is a mapping such that*:

$$M:P\to F^{℧}$$

*F*^℧^ *represents the collection of all Fuzzy subsets of* ℧ *and* $\langle \dot{e},M\left(\dot{e}\right)\rangle$ *is an ordered pair where* $\dot{e}$ *are the attributes from set* P⊆Ë, $M\left(\dot{e}\right)=\left\{\langle \varrho ,\mu_{F}\left(\varrho \right)\rangle |\varrho \in ℧\right\}$ *where* *μ*_*F*_(*ϱ*) *represents the membership degree from 0 to 1*.

**Definition 1.3**. [47] *Suppose* ℧ *be a set of discourse. Ë be an attributive set with respect to* ℧ *and* P *the subset of Ë. Then Cubic Soft Set can be expressed as*:

$$M_{P}=\left\{\left\langle \dot{e},M\left(\dot{e}\right)\right\rangle |\dot{e}\in P,M\left(\dot{e}\right)\in C^{℧}\right\}$$

*where M is a mapping such that*:

$$M:P\to C^{℧}$$

*C*^℧^ *represents all cubic subsets of* ℧. $\langle \dot{e},M\left(\dot{e}\right)\rangle$ *is an ordered pair where* $\dot{e}$ *are the attributes from set* P⊆Ë.

$$M\left(\dot{e}\right)=\left\{\langle \varrho ,\left(\mu_{C}^{I}\left(\varrho \right),\mu_{C}\left(\varrho \right)\right)\rangle |\in ℧\right\}\;\text{where}\;\mu_{C}^{I}\left(\varrho \right)\in \left[0,1\right]\;\text{and}\;\mu_{C}\left(\varrho \right)\subseteq \left[0,1\right].$$

**Definition 1.4**. [48] *Assume that* ℧ *be a universe of discourse and P*_1_, *P*_2_, *P*_3_…*P*_*n*_ *are the attributive sets, where, p*_1_, *p*_2_, *p*_3_…*p*_*n*_ *is the set of sub-attributes of P*_1_, *P*_2_, *P*_3_…*P*_*n*_ *respectively, with condition P*_*s*_ ∩ *P*_*t*_ = *ϕ*, *and* *s* ≠ *t*, *for all* *s*, *t* ∈ {1, 2, 3, ‥, *n*}. *Then* $H_{P}$ *is called hypersoft set as*:

$$H_{P}=\left\{\left\langle \hat{p},H\left(\hat{p}\right)\right\rangle |\hat{p}\in P,H\left(\hat{p}\right)\in P^{℧}\right\}$$

*where is the mapping as*:

$$H:P\to P^{℧}$$

$P=P_{1}\times P_{2}\times P_{3}\ldots \times P_{n}$ *each element of* P *is in the form of n-tuple*, $\hat{p}\in P$ *and* *P*^℧^ *represents all the subsets of* ℧.

**Definition 1.5**. [48] *Assume that* ℧ *be a universe of discourse and P*_1_, *P*_2_, *P*_3_…*P*_*n*_ *are the attributive sets, where, p*_1_, *p*_2_, *p*_3_…*p*_*n*_ *is the set of sub-attributes of P*_1_, *P*_2_, *P*_3_…*P*_*n*_ *respectively, with condition P*_*s*_ ∩ *P*_*t*_ = *ϕ*, *and*, *s* ≠ *t* *all* *s*, *t* ∈ {1, 2, ‥, *n*}. *Then* $H_{P}$ *is called Fuzzy hypersoft set as*:

$$H_{P}=\left\{\left\langle \hat{p},H\left(\hat{p}\right)\right\rangle |\hat{p}\in P,H\left(\hat{p}\right)\in F^{℧}\right\}$$

*where H is the mapping*:

$$H:P\to F^{℧}$$

*and* $P=P_{1}\times P_{2}\times P_{3}\ldots \times P_{n}$ *each element of* P *is in the form of n-tuple*. *F*^℧^ *represents all the fuzzy subsets of* ℧ *and*:

$$H\left(\hat{p}\right)=\left\{\varrho ,\mu_{F}\left(\varrho \right)|\varrho \in ℧\right\}.$$

**Fig 1:**
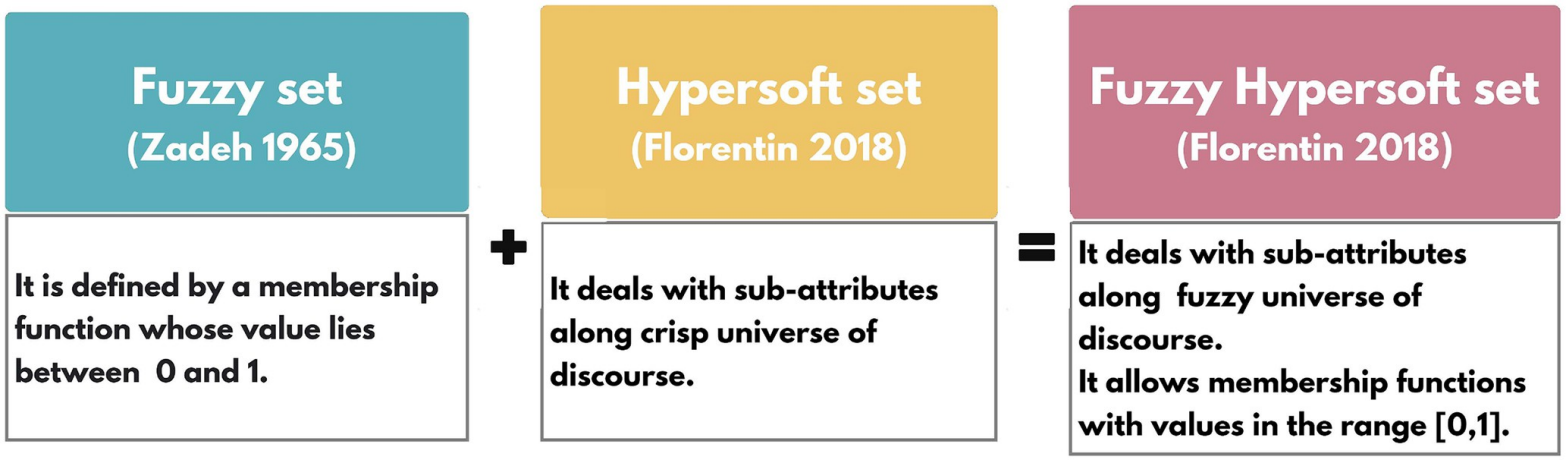
Fuzzy hypersoft set.

*A representation of the structure is provided in* Fig 1.

**Definition 1.6**. [48] *Assume that* ℧ *be a universe of discourse and P*_1_, *P*_2_, *P*_3_…*P*_*n*_ *are the attributive sets, where, p*_1_, *p*_2_, *p*_3_…*p*_*n*_ *is the set of sub-attributes of P*_1_, *P*_2_, *P*_3_…*P*_*n*_ *respectively, with condition P*_*s*_ ∩ *P*_*t*_ = *ϕ*, *and*, *s* ≠ *t all* *s*, *t* ∈ {1, 2, 3, ‥, *n*}. *Then* $H_{P}$ *is called Intuitionistic Fuzzy hypersoft set as*:

$$H_{P}=\left\{\left\langle \hat{p},H\left(\hat{p}\right)\right\rangle |\hat{p}\in P,H\left(\hat{p}\right)\in IF^{℧}\right\}$$

*where H is the mapping as*:

$$H:P\to IF^{℧}$$

*and* $P=P_{1}\times P_{2}\times P_{3}\ldots \times P_{n}$ *each element of* P *is in the form of n-tuple*. *IF*^℧^ *represents all the intuitionistic fuzzy subsets of* ℧.

*and*

$$H\left(\hat{p}\right)=\left\{\varrho ,\mu_{IF}\left(\varrho \right),\lambda_{IF}\left(\varrho \right)\right\}$$

*with*

$$0\leq \mu_{IF}+\lambda_{IF}\leq 1.$$

*If*

$$\begin{array}{c} \pi_{IF}\left(\varrho \right)=1-\mu_{IF}\left(\varrho \right)-\lambda_{IF}\left(\varrho \right) \end{array}$$

**Fig 2:**
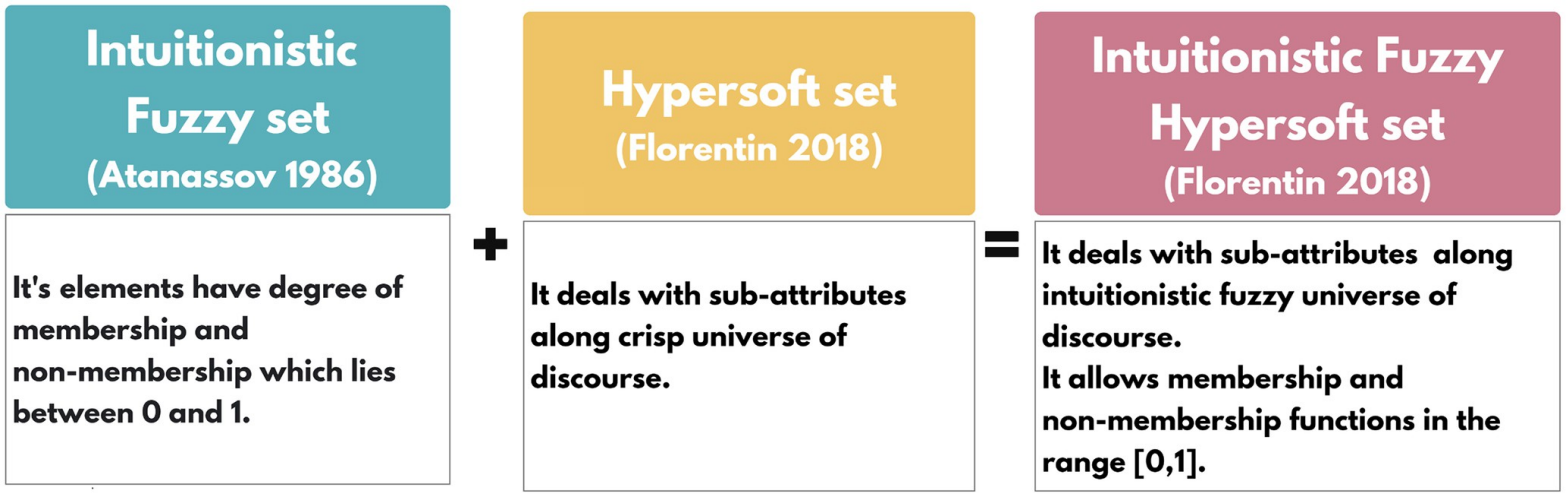
Intuitionistic fuzzy hypersoft set.

*Then*, *π*_*IF*_(*ϱ*) *is the degree of non-determinacy of the membership of the element ϱ* ∈ ℧ *to the set. A representation of the described structure is provided in* Fig 2.

## 3 Cubic intuitionistic fuzzy hypersoft set

**Definition 2.1**. *Assume that* ℧ *be a universe of discourse. Ë be an attributive set with respect to* ℧ *and* P *is the subset of Ë. Then Cubic Intuitionistic Fuzzy Soft Set can be expressed as*

$$M_{P}=\left\{\left\langle \dot{e},M\left(\dot{e}\right)\right\rangle |\dot{e}\in P,M\left(\dot{e}\right)\in CIF^{℧}\right\}$$

*where M is a mapping such as*:

$$M:P\to CIF^{℧}$$

*CIF*^℧^ *represents the collection of all cubic intuitionistic fuzzy subsets of* ℧.

$M\left(\dot{e}\right)=\left\{\langle \varrho ,\mu_{F}\left(\varrho \right),\lambda_{F}\left(\varrho \right)\rangle |\varrho \in ℧\right\}$ *where* $\mu_{F}\left(\varrho \right)$ *is an interval-valued intuitionistic fuzzy set and* λ_*F*_(*ϱ*) *is an intuitionistic fuzzy set. If*:

$$\begin{array}{c} \pi_{F}\left(\varrho \right)=1-\mu_{F}\left(\varrho \right)-\lambda_{F}\left(\varrho \right) \end{array}$$

*Then*, *π*_*F*_(*ϱ*) *is the degree of non-determinacy of the membership of the element* *ϱ* ∈ ℧ *to the set*.

**Fig 3:**
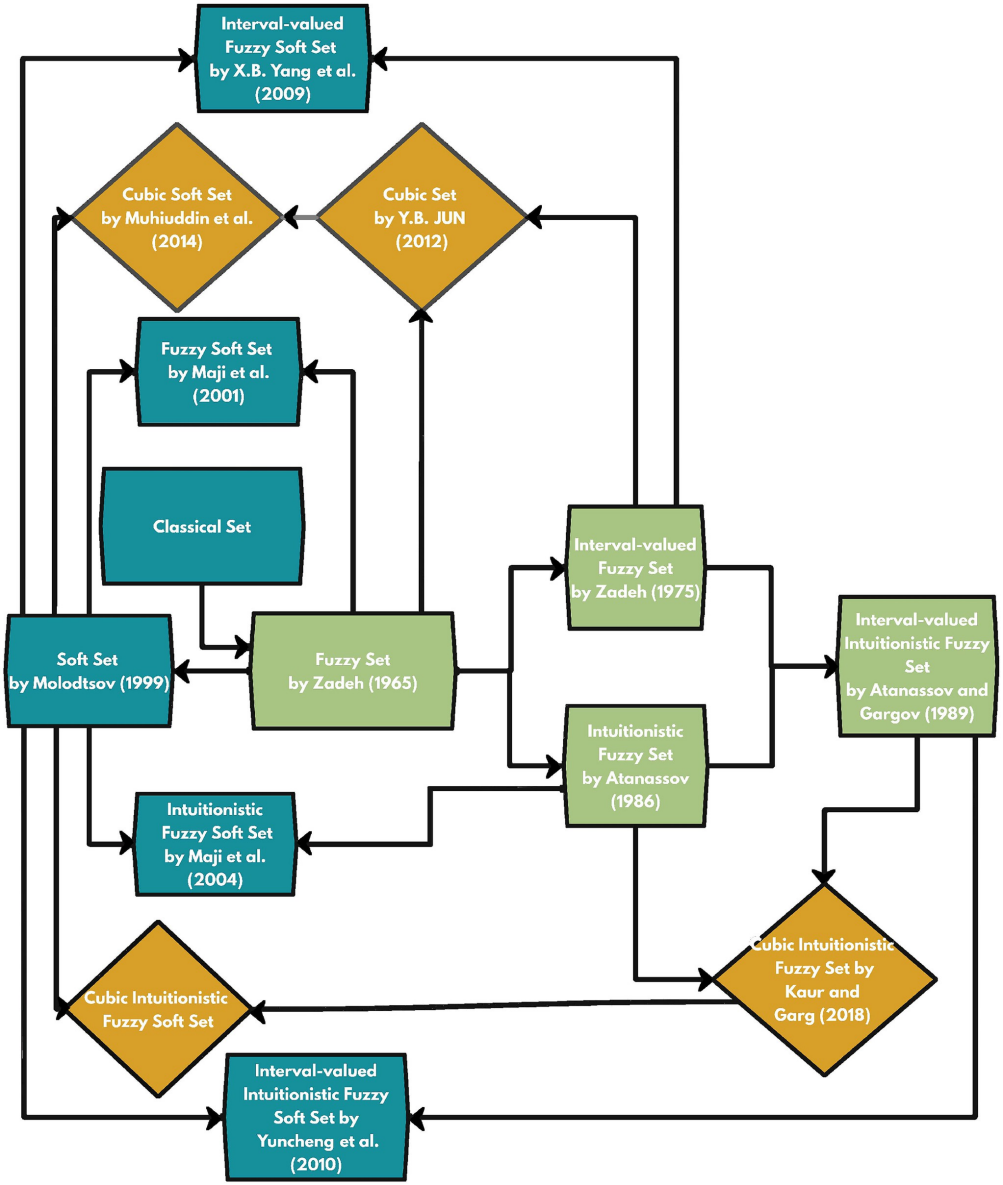
A Graphical Representation of the timeline of the developed structure.

Fig 3 provides a timeline of the development of fuzzy and soft hybrid structures over time.

**Definition 2.2**. *Assume that* ℧ *be a universe of discourse and P*_1_, *P*_2_, *P*_3_…*P*_*n*_ *are the sets of attributes, where*, *p*_1_, *p*_2_, *p*_3_…*p*_*n*_ *is the set of sub-attributes of* *P*_1_, *P*_2_, *P*_3_…*P*_*n*_ *respectively, with condition P*_*s*_ ∩ *P*_*t*_ = *ϕ*, *and*, *s* ≠ *t* *all* *s*, *t* ∈ {1, 2, 3, ‥, *n*}. *Then* $H_{P}$ *is called Cubic Intuitionistic Fuzzy hypersoft set as*:

$$H_{P}=\left\{\left\langle \hat{p},H\left(\hat{p}\right)\right\rangle |\hat{p}\in P,H\left(\hat{p}\right)\in CIF^{℧}\right\}$$

*where H is the mapping as*:

$$H:P\to CIF^{℧}$$

*and* $P=P_{1}\times P_{2}\times P_{3}\ldots \times P_{n}$ *each element of* P *is in the form of n-tuple*. *CIF*^℧^ *represents all the fuzzy subsets of* ℧.

*And*

$$H_{p}=\left\{\left\langle \varrho ,\left(\mu_{F}\left(\varrho \right),\lambda_{F}\left(\varrho \right)\right)\right\rangle |\varrho \in ℧\right\}$$

*where* (*μ*_F_(*ϱ*)) *is an interval-valued intuitionistic fuzzy set illustrated as*:

$$\left\{\left\langle \varrho ,\left(\mu_{IVIF}\left(\varrho \right),\lambda_{IVIF}\left(\varrho \right)\right)\right\rangle |\varrho \in ℧\right\}$$

*where*

$$\mu_{IVIF}\left(\varrho \right)\subseteq \left[0,1\right],\lambda_{IVIF}\left(\varrho \right)\subseteq \left[0,1\right]$$

*and* λ_*F*_(*ϱ*) *is an intuitionistic fuzzy set as*

$$\left\{\left\langle \varrho ,\left(\mu_{IF}\left(\varrho \right),\lambda_{IF}\left(\varrho \right)\right)\right\rangle |\varrho \in ℧\right\}$$

*where* *μ*_*IF*_(*ϱ*) ∈ [0, 1], λ_*IF*_(*ϱ*) ∈ [0, 1] *is a cubic intuitionistic fuzzy set, for all ϱ* ∈ ℧.

*If*

$$\begin{array}{c} \pi_{F}\left(\varrho \right)=1-\mu_{F}\left(\varrho \right)-\lambda_{F}\left(\varrho \right) \end{array}$$

**Fig 4:**
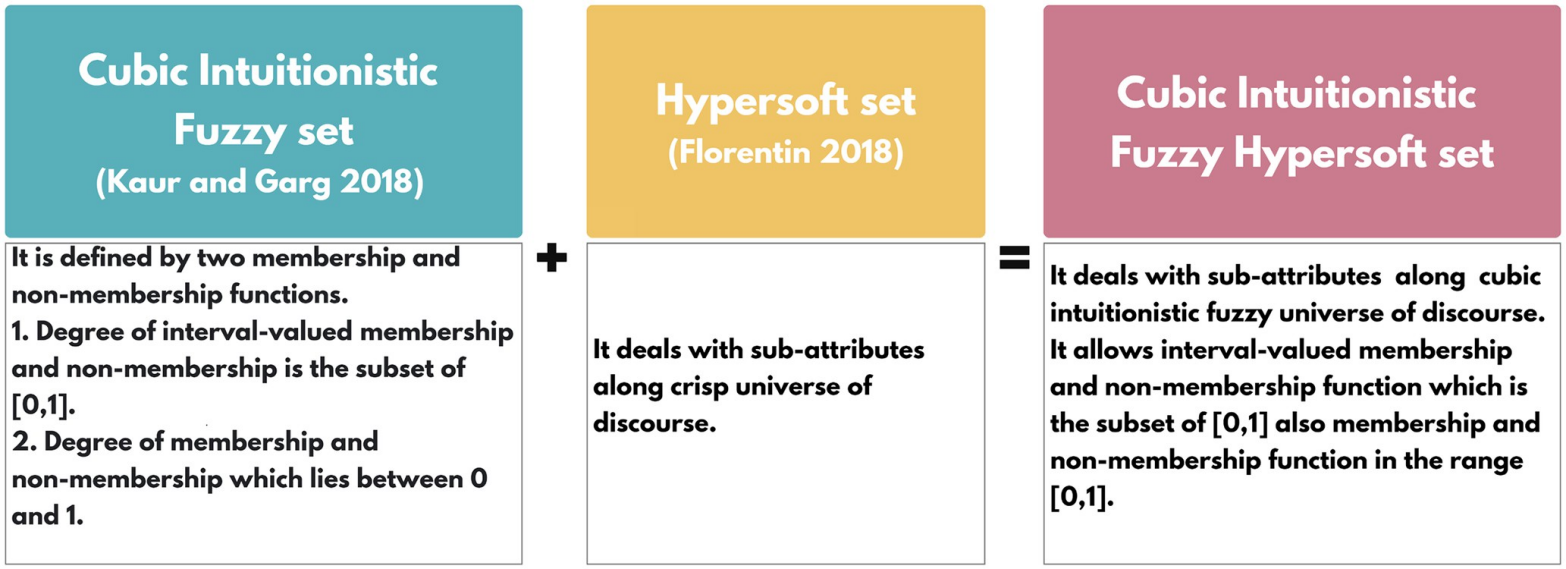
Cubic intuitionistic fuzzy hypersoft set.

*Then, π*_*F*_(*ϱ*) *is the degree of non-determinacy of the membership of the element* *ϱ* ∈ ℧ *to the set. The representation of the designed structure is provided in* Fig 4.

**Definition 2.3**. *Let* ℧ *be a universal set then* $H_{P}=\langle \hat{p},H\left(\hat{p}\right)\rangle$ *is said to be an internal cubic intuitionistic fuzzy hypersoft set if*:

$$\begin{array}{c} \mu_{\left(IF\right)}\left(\varrho \right)\in \mu_{\left(IVIF\right)}\left(\varrho \right)\;and\;\lambda_{\left(IF\right)}\left(\varrho \right)\in \lambda_{\left(IVIF\right)}\left(\varrho \right) \end{array}$$

*where μ*_(*IVIF*)_ *is a membership interval which is a subset of [0, 1] and* λ_(*IVIF*)_ *is a non-membership interval which is also a subset of [0, 1], for all* *ϱ* ∈ ℧.

**Definition 2.4**. *Let* ℧ *be a universal set then* $H_{P}=\langle \hat{p},H\left(\hat{p}\right)\rangle$ *is said to be an external cubic intuitionistic fuzzy hypersoft set if*:

$$\begin{array}{c} \mu_{\left(IF\right)}\left(\varrho \right)\notin \mu_{\left(IVIF\right)}\left(\varrho \right)\;and\;\lambda_{\left(IF\right)}\left(\varrho \right)\notin \lambda_{\left(IVIF\right)}\left(\varrho \right) \end{array}$$

**Definition 2.5**. *Let* ℧ *be a universal set then* $H_{P}=\langle \hat{p},H\left(\hat{p}\right)\rangle$ *is said to be a partial internal CIFS set if*:

$$\begin{array}{c} \mu_{\left(IF\right)}\left(\varrho \right)\in \mu_{\left(IVIF\right)}\left(\varrho \right)\;and\;\lambda_{\left(IF\right)}\left(\varrho \right)\notin \lambda_{\left(IVIF\right)}\left(\varrho \right) \end{array}$$

*or*

$$\begin{array}{c} \mu_{\left(IF\right)}\left(\varrho \right)\notin \mu_{\left(IVIF\right)}\left(\varrho \right)\;and\;\lambda_{\left(IF\right)}\left(\varrho \right)\in \lambda_{\left(IVIF\right)}\left(\varrho \right). \end{array}$$

**Definition 2.6**. *Let* ℧ *be a universe of discouse*, $H_{P}=\langle \hat{p},\left[\mu^{L},\mu^{U}\right],\left[\lambda^{L},\lambda^{U}\right],\left(\mu ,\lambda \right)\rangle$ *be a CIFHS where* $\hat{p}\in P=\left\{P_{1}\times P_{2}\times P_{3},..,P_{n}\right\}$ *each element of* P *is in the form of n-tuple and P*_1_, *P*_2_, ‥, *P*_*n*_ *are the attributive sets, for all* *ϱ* ∈ ℧ *then its complement will be*:

$$H_{P}^{C}=\langle \hat{p},\left[\lambda^{L},\lambda^{U}\right],\left[\mu^{L},\mu^{U}\right],\left(\lambda ,\mu \right)\rangle$$

*where* [*μ*^*L*^, *μ*^*U*^] *is the membership interval*, [λ^*L*^, λ^*U*^] *is the non-membership interval and μ is the membership value*, λ *is the non-membership value*.

**Definition 2.7**. *Let* ℧ *be an universal set*, $H_{P}=\langle \hat{p},\left[\mu^{L},\mu^{U}\right],\left[\lambda^{L},\lambda^{U}\right],\left(\mu ,\lambda \right)\rangle$ *and* $G_{Q}=\langle \hat{p},\left[\mu^{L},\mu^{U}\right],\left[\lambda^{L},\lambda^{U}\right],\left(\mu ,\lambda \right)\rangle$ *be two CIFHSs then direct sum will be defined as*:

$$\begin{array}{c} H_{P}\;oplus\;G_{Q}=[1-\left(1-\mu_{H_{P}}^{L}\right)\left(1-\mu_{G_{Q}}^{L}\right),1-\left(1-\mu_{H_{P}}^{U}\right)\left(1-\mu_{G_{Q}}^{U}\right)],[\lambda_{H_{P}}^{L}\lambda_{G_{Q}}^{L},\lambda_{H_{P}}^{U}\lambda_{G_{Q}}^{U}],\\ \left(1-\left(1-\mu_{H_{P}}\right)\left(1-\mu_{G_{Q}}\right),\lambda_{H_{P}}\lambda_{G_{Q}}\right) \end{array}$$

**Definition 2.8**. *Let* ℧ *be an universal set*, $H_{P}=\langle \hat{p},\left[\mu^{L},\mu^{U}\right],\left[\lambda^{L},\lambda^{U}\right],\left(\mu ,\lambda \right)\rangle$ *and* $G_{Q}=\langle \hat{p},\left[\mu^{L},\mu^{U}\right],\left[\lambda^{L},\lambda^{U}\right],\left(\mu ,\lambda \right)\rangle$ *be two CIFHSs then direct product will be defined as*:

$$\begin{array}{c} H_{P}\;times\;G_{Q}=[\mu_{H_{P}}^{L}\mu_{G_{Q}}^{L},\mu_{H_{P}}^{U}\mu_{G_{Q}}^{U}],[1-\left(1-\lambda_{H_{P}}^{L}\right)\left(1-\lambda_{G_{Q}}^{L}\right),1-\left(1-\lambda_{H_{P}}^{U}\right)\left(1-\lambda_{G_{Q}}^{U}\right)],\\ \left(\mu_{H_{P}}\mu_{G_{Q}},1-\left(1-\lambda_{H_{P}}\right)\left(1-\lambda_{G_{Q}}\right)\right) \end{array}$$

**Definition 2.9**. *Let* ℧ *be an universal set*, $H_{P}=\langle \hat{p},\left[\mu^{L},\mu^{U}\right],\left[\lambda^{L},\lambda^{U}\right],\left(\mu ,\lambda \right)\rangle$ *be a CIFHS and* S≥0 *be any real number. Then*:

$$\begin{array}{c} SH_{P}=[1-{\left(1-\mu^{L}\right)}^{S},1-{\left(1-\mu^{U}\right)}^{S}],[{\left(\lambda^{L}\right)}^{S},{\left(\lambda^{U}\right)}^{S}],(1-{\left(1-\mu \right)}^{S},{\left(\lambda \right)}^{S}) \end{array}$$

## 4 Development of distance measures using cubic intuitionistic fuzzy hypersoft set

Fuzzy distance measures are a type of distance metric used in fuzzy set theory to measure the similarity or dissimilarity between two fuzzy sets. There are several types of fuzzy distance measures, including Hamming distance, Euclidean distance, Jaccard distance, and Minkowski distance. These measures have been used in various applications, such as pattern recognition, medical diagnosis, and image processing [49–51]. A fuzzy distance measurement known as the hamming distance counts the number of locations where two fuzzy sets have distinct membership values. Fuzzy In many different applications, including data mining, image processing, and bioinformatics, the hamming distance is a widely used distance metric. The degree of membership of items in a fuzzy set is taken into account in this fuzzy variation of the traditional Hamming distance.

Fuzzy Hamming distance has been used in bioinformatics to gauge how similar DNA sequences are to one another. To compare the DNA sequences of two closely related bacterial strains, researchers used fuzzy hamming distance. They discovered that Fuzzy Hamming distance could detect minute variations between the strains that traditional Hamming distance was unable to [52]. The field of image processing has also used fuzzy Hamming distance. Fuzzy Hamming distance was used to compare the features of two faces using the metric in the development of a facial recognition system. The researchers discovered that Fuzzy Hamming distance has a high degree of accuracy for classifying the two faces [53].

Fuzzy Hamming distance has been used in literature a number of times for the comparison of multiple-attribute alternatives where normal hamming distance falls short. In this study, with a number of examples found in literature for computing different types of distance measures, we propose 6 different forms of Hamming distances for development of a pattern recognition system for quality control.

### 4.1 Hamming distance measures

A number of Hamming distances are developed to cover a range of scenarios using a variety of methods for manipulation of data to reach the desired outcome. The developed distance measures are as follows:

#### 4.1.1 Hamming distance measure

The Hamming distance measure using CIFHSs is defined as:

$$\begin{array}{c} d_{1}\left(H_{P},G_{Q}\right)=\frac{1}{6m}\sum_{i=1}^{m}\sum_{j=1}^{n}(\begin{array}{c} {\hat{p}}_{i}|\mu_{H_{P}}^{L}\left(\varrho_{j}\right)-\mu_{G_{Q}}^{L}\left(\varrho_{j}\right)|+{\hat{p}}_{i}\left|\mu_{H_{P}}^{U}\left(\varrho_{j}\right)-\mu_{G_{Q}}^{U}\left(\varrho_{j}\right)\right|+\\ {\hat{p}}_{i}|\lambda_{H_{P}}^{L}\left(\varrho_{j}\right)-\lambda_{G_{Q}}^{L}\left(\varrho_{j}\right)|+{\hat{p}}_{i}\left|\lambda_{H_{P}}^{U}\left(\varrho_{j}\right)-\lambda_{G_{Q}}^{U}\left(\varrho_{j}\right)\right|+\\ {\hat{p}}_{i}|\mu_{H_{P}}\left(\varrho_{j}\right)-\mu_{G_{Q}}\left(\varrho_{j}\right)|+{\hat{p}}_{i}\left|\lambda_{H_{P}}\left(\varrho_{j}\right)-\lambda_{G_{Q}}\left(\varrho_{j}\right)\right| \end{array}) \end{array}$$

Here *ϱ* are the elements of ℧ which is a universal set, “n” represents the number of elements of universal sets and “m” represents the number of attributes.

**Theorem 3.1**. *Distance d*_1_($H_{P}$,$G_{Q}$) *is said to be distance measure if it satisfies the following properties*:

1.
$0\leq d_{1}(H_{P}$ ,$G_{Q})\leq 1$
2.
$d_{1}(H_{P},G_{Q})=0$ *if and only if* $H_{P}=G_{Q}$

3.
$d_{1}(H_{P},G_{Q})=d_{1}(G_{Q},H_{P})$

4. *If* $H_{P}\subseteq G_{Q}\subseteq I_{R}$ , *then* $d_{1}(H_{P},G_{Q})\leq d_{1}(H_{P},I_{R})$ *and* $d_{1}(G_{Q},I_{R})\leq d_{1}(H_{P},I_{R}$).

*Proof*. For two CIFHSs $H_{P}=\langle \hat{p},\left[\mu_{H_{P}}^{L},\mu_{H_{P}}^{U}\right],\left[\lambda_{H_{P}}^{L},\lambda_{H_{P}}^{U}\right],\left(\mu_{H_{P}},\lambda_{H_{P}}\right)\rangle$ and $G_{Q}=\langle \hat{p},\left[\mu_{G_{Q}}^{L},\mu_{G_{Q}}^{U}\right],\left[\lambda_{G_{Q}}^{L},\lambda_{G_{Q}}^{U}\right],\left(\mu_{G_{Q}},\lambda_{G_{Q}}\right)\rangle$, We have

**1)** By the definition of distance, it is that $d_{1}(H_{P},G_{Q})\geq 0$. For it to be valid, $d_{1}(H_{P},G_{Q})$ ≤ must be 1. By using the definition of CIFHSs the membership and non-membership values belongs to [0, 1],s

Such that 0 $\leq \mu_{H_{P}}^{L}\left(\varrho_{j}\right),\mu_{H_{P}}^{U}\left(\varrho_{j}\right)\leq 1,0\leq \lambda_{H_{P}}^{L}\left(\varrho_{j}\right),\lambda_{H_{P}}^{U}\left(\varrho_{j}\right)\leq 1$ and $0\leq \mu_{H_{P}}\left(\varrho_{j}\right),\lambda_{H_{P}}\left(\varrho_{j}\right)\leq 1,$

Similarly, 0 $\leq \mu_{G_{Q}}^{L}\left(\varrho_{j}\right),\mu_{G_{Q}}^{U}\left(\varrho_{j}\right)\leq 1$, $0\leq \lambda_{G_{Q}}^{L}\left(\varrho_{j}\right),\lambda_{G_{Q}}^{U}\left(\varrho_{j}\right)\leq 1$, and $0\leq \mu_{G_{Q}}\left(\varrho_{j}\right),\lambda_{G_{Q}}\left(\varrho_{j}\right)\leq 1$.

This implies that:

$0\leq |\mu_{H_{P}}^{L}\left(\varrho_{j}\right)-\mu_{G_{Q}}^{L}\left(\varrho_{j}\right)|\leq 1$ , $0\leq |\mu_{H_{P}}^{U}\left(\varrho_{j}\right)-\mu_{G_{Q}}^{U}\left(\varrho_{j}\right)|\leq 1$, $0|\lambda_{H_{P}}^{L}\left(\varrho_{j}\right)-\lambda_{G_{Q}}^{L}\left(\varrho_{j}\right)|\leq 1$ and $0\leq |\lambda_{H_{P}}^{U}\left(\varrho_{j}\right)-\lambda_{G_{Q}}^{U}\left(\varrho_{j}\right)|\leq 1$, $0\leq |\mu_{H_{P}}\left(\varrho_{j}\right)-\mu_{G_{Q}}\left(\varrho_{j}\right)|\leq 1$, $0\leq |\lambda_{H_{P}}\left(\varrho_{j}\right)-\lambda_{G_{Q}}\left(\varrho_{j}\right)|\leq 1$ for all i and j, then:

$$\begin{array}{c} 0\leq (\begin{array}{c} {\hat{p}}_{i}|\mu_{H_{P}}^{L}\left(\varrho_{j}\right)-\mu_{G_{Q}}^{L}\left(\varrho_{j}\right)|+{\hat{p}}_{i}\left|\mu_{H_{P}}^{U}\left(\varrho_{j}\right)-\mu_{G_{Q}}^{U}\left(\varrho_{j}\right)\right|+\\ {\hat{p}}_{i}|\lambda_{H_{P}}^{L}\left(\varrho_{j}\right)-\lambda_{G_{Q}}^{L}\left(\varrho_{j}\right)|+{\hat{p}}_{i}\left|\lambda_{H_{P}}^{U}\left(\varrho_{j}\right)-\lambda_{G_{Q}}^{U}\left(\varrho_{j}\right)\right|+\\ {\hat{p}}_{i}|\mu_{H_{P}}\left(\varrho_{j}\right)-\mu_{G_{Q}}\left(\varrho_{j}\right)|+{\hat{p}}_{i}\left|\lambda_{H_{P}}\left(\varrho_{j}\right)-\lambda_{G_{Q}}\left(\varrho_{j}\right)\right| \end{array})\leq 6 \end{array}$$

⇒ $0\leq d_{1}(H_{P}$,$G_{Q})\leq 1$

**2)** Let $d_{1}(H_{P},G_{Q})=0$, For two CIFHSs $H_{P}$ and $G_{Q}$,

$$\Rightarrow \frac{1}{6m}\sum_{i=1}^{m}\sum_{j=1}^{n}(\begin{array}{c} {\hat{p}}_{i}|\mu_{H_{P}}^{L}\left(\varrho_{j}\right)-\mu_{G_{Q}}^{L}\left(\varrho_{j}\right)|+{\hat{p}}_{i}\left|\mu_{H_{P}}^{U}\left(\varrho_{j}\right)-\mu_{G_{Q}}^{U}\left(\varrho_{j}\right)\right|+\\ {\hat{p}}_{i}|\lambda_{H_{P}}^{L}\left(\varrho_{j}\right)-\lambda_{G_{Q}}^{L}\left(\varrho_{j}\right)|+{\hat{p}}_{i}\left|\lambda_{H_{P}}^{U}\left(\varrho_{j}\right)-\lambda_{G_{Q}}^{U}\left(\varrho_{j}\right)\right|+\\ {\hat{p}}_{i}|\mu_{H_{P}}\left(\varrho_{j}\right)-\mu_{G_{Q}}\left(\varrho_{j}\right)|+{\hat{p}}_{i}\left|\lambda_{H_{P}}\left(\varrho_{j}\right)-\lambda_{G_{Q}}\left(\varrho_{j}\right)\right| \end{array})=0$$

If and only if, For all j, $|\mu_{H_{P}}^{L}\left(\varrho_{j}\right)-\mu_{G_{Q}}^{L}\left(\varrho_{j}\right)\left|=0,\right|\mu_{H_{P}}^{U}\left(\varrho_{j}\right)-\mu_{G_{Q}}^{U}\left(\varrho_{j}\right)\left|=0,\right|\lambda_{H_{P}}^{L}\left(\varrho_{j}\right)-\lambda_{G_{Q}}^{L}\left(\varrho_{j}\right)\left|=0,\right|\lambda_{H_{P}}^{U}\left(\varrho_{j}\right)-\lambda_{G_{Q}}^{U}\left(\varrho_{j}\right)\left|=0,\right|\mu_{H_{P}}\left(\varrho_{j}\right)-\mu_{G_{Q}}\left(\varrho_{j}\right)\left|=0,\right|\lambda_{H_{P}}\left(\varrho_{j}\right)-\lambda_{G_{Q}}\left(\varrho_{j}\right)|=0$ which is equivalent to $\mu_{H_{P}}^{L}\left(\varrho_{j}\right)=\mu_{G_{Q}}^{L}\left(\varrho_{j}\right),\mu_{H_{P}}^{U}\left(\varrho_{j}\right)=\mu_{G_{Q}}^{U}\left(\varrho_{j}\right),\lambda_{H_{P}}^{L}\left(\varrho_{j}\right)=\lambda_{G_{Q}}^{L}\left(\varrho_{j}\right),\lambda_{H_{P}}^{U}\left(\varrho_{j}\right)=\lambda_{G_{Q}}^{U}\left(\varrho_{j}\right),\mu_{H_{P}}\left(\varrho_{j}\right)=\mu_{G_{Q}}\left(\varrho_{j}\right),\lambda_{H_{P}}\left(\varrho_{j}\right)=\lambda_{G_{Q}}\left(\varrho_{j}\right)$ Thus $d_{1}(H_{P},G_{Q})=0\Rightarrow H_{P}=G_{Q}$

**3)** For two CIFHSs $H_{P}$ and $G_{Q}$,

$$\begin{array}{cc} d_{1}\left(H_{P},G_{Q}\right) & =\frac{1}{6m}\sum_{i=1}^{m}\sum_{j=1}^{n}(\begin{array}{c} {\hat{p}}_{i}|\mu_{H_{P}}^{L}\left(\varrho_{j}\right)-\mu_{G_{Q}}^{L}\left(\varrho_{j}\right)|+{\hat{p}}_{i}\left|\mu_{H_{P}}^{U}\left(\varrho_{j}\right)-\mu_{G_{Q}}^{U}\left(\varrho_{j}\right)\right|+\\ {\hat{p}}_{i}|\lambda_{H_{P}}^{L}\left(\varrho_{j}\right)-\lambda_{G_{Q}}^{L}\left(\varrho_{j}\right)|+{\hat{p}}_{i}\left|\lambda_{H_{P}}^{U}\left(\varrho_{j}\right)-\lambda_{G_{Q}}^{U}\left(\varrho_{j}\right)\right|+\\ {\hat{p}}_{i}|\mu_{H_{P}}\left(\varrho_{j}\right)-\mu_{G_{Q}}\left(\varrho_{j}\right)|+{\hat{p}}_{i}\left|\lambda_{H_{P}}\left(\varrho_{j}\right)-\lambda_{G_{Q}}\left(\varrho_{j}\right)\right| \end{array})\\ & =\frac{1}{6m}\sum_{i=1}^{m}\sum_{j=1}^{n}(\begin{array}{c} {\hat{p}}_{i}|\mu_{G_{Q}}^{L}\left(\varrho_{j}\right)-\mu_{H_{P}}^{L}\left(\varrho_{j}\right)|+{\hat{p}}_{i}\left|\mu_{G_{Q}}^{U}\left(\varrho_{j}\right)-\mu_{H_{P}}^{U}\left(\varrho_{j}\right)\right|+\\ {\hat{p}}_{i}|\lambda_{G_{Q}}^{L}\left(\varrho_{j}\right)-\lambda_{H_{P}}^{L}\left(\varrho_{j}\right)|+{\hat{p}}_{i}\left|\lambda_{G_{Q}}^{U}\left(\varrho_{j}\right)-\lambda_{H_{P}}^{U}\left(\varrho_{j}\right)\right|+\\ {\hat{p}}_{i}|\mu_{G_{Q}}\left(\varrho_{j}\right)-\mu_{H_{P}}\left(\varrho_{j}\right)|+{\hat{p}}_{i}\left|\lambda_{G_{Q}}\left(\varrho_{j}\right)-\lambda_{H_{P}}\left(\varrho_{j}\right)\right| \end{array})\\ & =d_{1}(G_{Q},H_{P}) \end{array}$$

Hence $d_{1}(H_{P},G_{Q})=d_{1}(G_{Q},H_{P})$

**4)** If $H_{P}$ ⊆ $G_{Q}$ ⊆ $I_{R}$ then $\left[\mu_{H_{P}}^{L}\left(\varrho_{j}\right),\mu_{H_{P}}^{U}\left(\varrho_{j}\right)\right]\subseteq \left[\mu_{G_{Q}}^{L}\left(\varrho_{j}\right),\mu_{G_{Q}}^{U}\left(\varrho_{j}\right)\right]\subseteq \left[\mu_{I_{R}}^{L}\left(\varrho_{j}\right),\mu_{I_{R}}^{U}\left(\varrho_{j}\right)\right]$, $\left[\lambda_{H_{P}}^{L}\left(\varrho_{j}\right),\lambda_{H_{P}}^{U}\left(\varrho_{j}\right)\right]⊇\left[\lambda_{G_{Q}}^{L}\left(\varrho_{j}\right),\lambda_{G_{Q}}^{U}\left(\varrho_{j}\right)\right]⊇\left[\lambda_{I_{R}}^{L}\left(\varrho_{j}\right),\lambda_{I_{R}}^{U}\left(\varrho_{j}\right)\right]$, Also $\mu_{H_{P}}\left(\varrho_{j}\right)\geq \mu_{G_{Q}}\left(\varrho_{j}\right)\geq \mu_{I_{R}}\left(\varrho_{j}\right)$ and $\lambda_{H_{P}}\left(\varrho_{j}\right)\leq \lambda_{G_{Q}}\left(\varrho_{j}\right)\leq \lambda_{I_{R}}\left(\varrho_{j}\right)$.

Therefore, $|\mu_{H_{P}}^{L}\left(\varrho_{j}\right),\mu_{G_{Q}}^{L}\left(\varrho_{j}\right)|\leq |\mu_{H_{P}}^{L}\left(\varrho_{j}\right),\mu_{I_{R}}^{L}\left(\varrho_{j}\right)|,|\mu_{H_{P}}^{U}\left(\varrho_{j}\right),\mu_{G_{Q}}^{U}\left(\varrho_{j}\right)|\leq |\mu_{H_{P}}^{U}\left(\varrho_{j}\right),\mu_{I_{R}}^{U}\left(\varrho_{j}\right)|$, $|\lambda_{H_{P}}^{L}\left(\varrho_{j}\right),\lambda_{G_{Q}}^{L}\left(\varrho_{j}\right)|\leq |\lambda_{H_{P}}^{L}\left(\varrho_{j}\right),\lambda_{I_{R}}^{L}\left(\varrho_{j}\right)|,|\lambda_{H_{P}}^{U}\left(\varrho_{j}\right),\lambda_{G_{Q}}^{U}\left(\varrho_{j}\right)|\leq |\lambda_{H_{P}}^{U}\left(\varrho_{j}\right),\lambda_{I_{R}}^{U}\left(\varrho_{j}\right)|$, $|\mu_{H_{P}}\left(\varrho_{j}\right),\mu_{G_{Q}}\left(\varrho_{j}\right)|\leq |\mu_{H_{P}}\left(\varrho_{j}\right),\mu_{I_{R}}\left(\varrho_{j}\right)|$ and $|\lambda_{H_{P}}\left(\varrho_{j}\right),\lambda_{G_{Q}}\left(\varrho_{j}\right)|\leq |\lambda_{H_{P}}\left(\varrho_{j}\right),\lambda_{I_{R}}\left(\varrho_{j}\right)|$.

Thus,

$$\begin{array}{cc} d_{1}\left(H_{P},I_{R}\right) & =\frac{1}{6m}\sum_{i=1}^{m}\sum_{j=1}^{n}(\begin{array}{c} {\hat{p}}_{i}|\mu_{H_{P}}^{L}\left(\varrho_{j}\right)-\mu_{I_{R}}^{L}\left(\varrho_{j}\right)|+{\hat{p}}_{i}\left|\mu_{H_{P}}^{U}\left(\varrho_{j}\right)-\mu_{I_{R}}^{U}\left(\varrho_{j}\right)\right|+\\ {\hat{p}}_{i}|\lambda_{H_{P}}^{L}\left(\varrho_{j}\right)-\lambda_{I_{R}}^{L}\left(\varrho_{j}\right)|+{\hat{p}}_{i}\left|\lambda_{H_{P}}^{U}\left(\varrho_{j}\right)-\lambda_{I_{R}}^{U}\left(\varrho_{j}\right)\right|+\\ {\hat{p}}_{i}|\mu_{H_{P}}\left(\varrho_{j}\right)-\mu_{I_{R}}\left(\varrho_{j}\right)|+{\hat{p}}_{i}\left|\lambda_{H_{P}}\left(\varrho_{j}\right)-\lambda_{I_{R}}\left(\varrho_{j}\right)\right| \end{array})\\ & \geq \frac{1}{6m}\sum_{i=1}^{m}\sum_{j=1}^{n}(\begin{array}{c} {\hat{p}}_{i}|\mu_{H_{P}}^{L}\left(\varrho_{j}\right)-\mu_{G_{Q}}^{L}\left(\varrho_{j}\right)|+{\hat{p}}_{i}\left|\mu_{H_{P}}^{U}\left(\varrho_{j}\right)-\mu_{G_{Q}}^{U}\left(\varrho_{j}\right)\right|+\\ {\hat{p}}_{i}|\lambda_{H_{P}}^{L}\left(\varrho_{j}\right)-\lambda_{G_{Q}}^{L}\left(\varrho_{j}\right)|+{\hat{p}}_{i}\left|\lambda_{H_{P}}^{U}\left(\varrho_{j}\right)-\lambda_{G_{Q}}^{U}\left(\varrho_{j}\right)\right|+\\ {\hat{p}}_{i}|\mu_{H_{P}}\left(\varrho_{j}\right)-\mu_{G_{Q}}\left(\varrho_{j}\right)|+{\hat{p}}_{i}\left|\lambda_{H_{P}}\left(\varrho_{j}\right)-\lambda_{G_{Q}}\left(\varrho_{j}\right)\right| \end{array})\\ & =d_{1}(H_{P},G_{Q}) \end{array}$$

Similarly, $d_{1}(H_{P},I_{R})\geq d_{1}(G_{Q},I_{R})$.

Hence, *d*_1_ is a valid distance measure.

#### 4.1.2 Normalized hamming distance measure

The normalized hamming distance measure can be defined as:

$$\begin{array}{c} d_{2}\left(H_{P},G_{Q}\right)=\frac{1}{6mn}\sum_{i=1}^{m}\sum_{j=1}^{n}(\begin{array}{c} {\hat{p}}_{i}|\mu_{H_{P}}^{L}\left(\varrho_{j}\right)-\mu_{G_{Q}}^{L}\left(\varrho_{j}\right)|+{\hat{p}}_{i}\left|\mu_{H_{P}}^{U}\left(\varrho_{j}\right)-\mu_{G_{Q}}^{U}\left(\varrho_{j}\right)\right|+\\ {\hat{p}}_{i}|\lambda_{H_{P}}^{L}\left(\varrho_{j}\right)-\lambda_{G_{Q}}^{L}\left(\varrho_{j}\right)|+{\hat{p}}_{i}\left|\lambda_{H_{P}}^{U}\left(\varrho_{j}\right)-\lambda_{G_{Q}}^{U}\left(\varrho_{j}\right)\right|+\\ {\hat{p}}_{i}|\mu_{H_{P}}\left(\varrho_{j}\right)-\mu_{G_{Q}}\left(\varrho_{j}\right)|+{\hat{p}}_{i}\left|\lambda_{H_{P}}\left(\varrho_{j}\right)-\lambda_{G_{Q}}\left(\varrho_{j}\right)\right| \end{array}) \end{array}$$

here *ϱ* are the elements of ℧ which is a universal set, “n” represents the number of elements of universal sets and “m” represents the number of attributes.

**Theorem 3.2**. *Distance d*_1_($H_{P}$, $G_{Q}$) *is said to be distance measure if it satisfies the following properties*:

1.
$0\leq d_{1}(H_{P},G_{Q})\leq 1$

2. $d_{1}(H_{P},G_{Q})=0$ *if and only if* $H_{P}=G_{Q}$
3.
$d_{1}(H_{P},G_{Q})=d_{1}(G_{Q},H_{P})$

4. *If* $H_{P}\subseteq G_{Q}\subseteq I_{R}$, *then* $d_{1}(H_{P},G_{Q})\leq d_{1}(H_{P},I_{R})$ *and* $d_{1}(G_{Q},I_{R})\leq d_{1}(H_{P},I_{R})$.

#### 4.1.3 Weighted hamming distance measure

The weighted hamming distance for CIFHSS is defined as:

$$\begin{array}{c} d_{3}\left(H_{P},G_{Q}\right)=\frac{1}{6m}\sum_{i=1}^{m}\sum_{j=1}^{n}C_{i}(\begin{array}{c} F\left(e_{i}\right)|\mu_{H_{P}}^{L}\left(\varrho_{j}\right)-\mu_{G_{Q}}^{L}\left(\varrho_{j}\right)|+F\left(e_{i}\right)\left|\mu_{H_{P}}^{U}\left(\varrho_{j}\right)-\mu_{G_{Q}}^{U}\left(\varrho_{j}\right)\right|+\\ F\left(e_{i}\right)|\lambda_{H_{P}}^{L}\left(\varrho_{j}\right)-\lambda_{G_{Q}}^{L}\left(\varrho_{j}\right)|+F\left(e_{i}\right)\left|\lambda_{H_{P}}^{U}\left(\varrho_{j}\right)-\lambda_{G_{Q}}^{L}\left(\varrho_{j}\right)\right|+\\ F\left(e_{i}\right)|\mu_{H_{P}}\left(\varrho_{j}\right)-\mu_{G_{Q}}\left(\varrho_{j}\right)|+F\left(e_{i}\right)\left|\lambda_{H_{P}}\left(\varrho_{j}\right)-\lambda_{G_{Q}}\left(\varrho_{j}\right)\right| \end{array}) \end{array}$$ (1)

Here, *ϱ* are the elements of ℧ which is a universal set, “n” represents the number of elements of universal sets and “m” represents the number of attributes.

### 4.2 Hausdorff distance measures

The Hausdorff distance measure can be calculated using the formula below: Let $H_{P}$, $G_{Q}$ are CIFHSs then Hausdorff distance measures will discussed as

#### 4.2.1 Hausdorff hamming distance measure

$$\begin{array}{c} d_{1}^{H}\left(H_{P},G_{Q}\right)=\frac{1}{6m}\sum_{i=1}^{m}\sum_{j=1}^{n}(\begin{array}{c} max(\begin{array}{c} F\left(e_{i}\right)|\mu_{H_{P}}^{L}\left(\varrho_{j}\right)-\mu_{G_{Q}}^{L}\left(\varrho_{j}\right)|,F\left(e_{i}\right)\left|\mu_{H_{P}}^{U}\left(\varrho_{j}\right)-\mu_{G_{Q}}^{U}\left(\varrho_{j}\right)\right|,\\ F\left(e_{i}\right)|\lambda_{H_{P}}^{L}\left(\varrho_{j}\right)-\lambda_{G_{Q}}^{L}\left(\varrho_{j}\right)|,F\left(e_{i}\right)\left|\lambda_{H_{P}}^{U}\left(\varrho_{j}\right)-\lambda_{G_{Q}}^{U}\left(\varrho_{j}\right)\right|,\\ F\left(e_{i}\right)|\mu_{H_{P}}\left(\varrho_{j}\right)-\mu_{G_{Q}}\left(\varrho_{j}\right)|,F\left(e_{i}\right)\left|\lambda_{H_{P}}\left(\varrho_{j}\right)-\lambda_{G_{Q}}\left(\varrho_{j}\right)\right| \end{array}) \end{array}) \end{array}$$

Here *ϱ* are the elements of ℧ which is a universal set, “n” represents the number of elements of universal sets and “m” represents the number of attributes.

**Theorem 3.3** *Distance* $d_{1}^{H}\left(H_{P},G_{Q}\right)$ *is said to be distance measure if it satisfies the following properties*:

1.
$0\leq d_{1}^{H}\left(H_{P},G_{Q}\right)\leq 1$

2.
$d_{1}^{H}\left(H_{P},G_{Q}\right)=0\Leftrightarrow H_{P}=G_{Q}$

3.
$d_{1}^{H}\left(H_{P},G_{Q}\right)=d_{1}^{H}\left(G_{Q},H_{P}\right))$

4. $IfH_{P}\subseteq G_{Q}\subseteq I_{R},\;then\;d_{1}^{H}\left(H_{P},G_{Q}\right)\leq d_{1}^{H}\left(H_{P},I_{R}\right)\;and\;d_{1}^{H}\left(G_{Q},I_{R}\right)\leq d_{1}^{H}\left(H_{P},I_{R}\right)$.

*Proof*. For two CIFHSs $H_{P}=\langle F\left(e_{i}\right),\left[\mu_{H_{P}}^{L},\mu_{H_{P}}^{U}\right],\left[\lambda_{H_{P}}^{L},\lambda_{H_{P}}^{U}\right],\left(\mu_{H_{P}},\lambda_{H_{P}}\right)\rangle$ and $\langle F\left(e_{i}\right),\left[\mu_{G_{Q}}^{L},\mu_{G_{Q}}^{U}\right],\left[\lambda_{G_{Q}}^{L},\lambda_{G_{Q}}^{U}\right],\left(\mu_{G_{Q}},\lambda_{G_{Q}}\right)\rangle$, We have

**1)** By the definition of distance, it is that $d_{1}^{H}\left(H_{P},G_{Q}\right)\geq 0$. For it to be valid, $d_{1}^{H}\left(H_{P},G_{Q}\right)\geq 0\leq \;mustbe\;1$. By using the definition of CIFHSs the membership and non-membership values belongs to [0, 1]

Such that $0\leq max\left(\mu_{H_{P}}^{L}\left(\varrho_{j}\right)\right),max\left(\mu_{H_{P}}^{U}\left(\varrho_{j}\right)\right)\leq 1$,

$0\leq max\left(\lambda_{H_{P}}^{L}\left(\varrho_{j}\right)\right),max\left(\lambda_{H_{P}}^{U}\left(\varrho_{j}\right)\right)\leq 1$ and $0\leq max\left(\mu_{H_{P}}\left(\varrho_{j}\right)\right),max\left(\lambda_{H_{P}}\left(\varrho_{j}\right)\right)\leq 1$,

Similarly,

$0\leq max\left(\mu_{G_{Q}}^{L}\left(\varrho_{j}\right)\right),max\left(\mu_{G_{Q}}^{U}\left(\varrho_{j}\right)\right)\leq 1$ ,

$0\leq max\left(\lambda_{G_{Q}}^{L}\left(\varrho_{j}\right)\right),max\left(\lambda_{G_{Q}}^{U}\left(\varrho_{j}\right)\right)\leq 1$ and $0\leq max\left(\mu_{G_{Q}}\left(\varrho_{j}\right)\right),max\left(\lambda_{G_{Q}}\left(\varrho_{j}\right)\right)\leq 1$,

This implies that:

$0\leq max|\mu_{H_{P}}^{L}\left(\varrho_{j}\right)-\mu_{G_{Q}}^{L}\left(\varrho_{j}\right)|\leq 1$ , $0\leq max|\mu_{H_{P}}^{U}\left(\varrho_{j}\right)-\mu_{G_{Q}}^{U}\left(\varrho_{j}\right)|\leq 1$, $0\leq max|\lambda_{H_{P}}^{L}\left(\varrho_{j}\right)-\lambda_{G_{Q}}^{L}\left(\varrho_{j}\right)|\leq 1$ and $0\leq max|\lambda_{H_{P}}^{U}\left(\varrho_{j}\right)-\lambda_{G_{Q}}^{U}\left(\varrho_{j}\right)|\leq 1$, $0\leq max|\mu_{H_{P}}\left(\varrho_{j}\right)-\mu_{G_{Q}}\left(\varrho_{j}\right)|\leq 1$, $0\leq max|\lambda_{H_{P}}\left(\varrho_{j}\right)-\lambda_{G_{Q}}\left(\varrho_{j}\right)|\leq 1$

or all *i* and *j*, then:

$$0\leq max\left(\begin{array}{l} F(e_{i})\left|\mu_{H_{\mathbb{P}}}^{L}(\varrho_{j})-\mu_{G_{\mathbb{Q}}}^{L}(\varrho_{j})\right|,F(e_{i})\left|\mu_{H_{\mathbb{P}}}^{U}(\varrho_{j})-\mu_{G_{\mathbb{Q}}}^{U}(\varrho_{j})\right|,\\ F(e_{i})\left|\lambda_{H_{\mathbb{P}}}^{L}(\varrho_{j})-\lambda_{G_{\mathbb{Q}}}^{L}(\varrho_{j})\right|,F(e_{i})\left|\lambda_{H_{\mathbb{P}}}^{U}(\varrho_{j})-\lambda_{G_{\mathbb{R}}}^{U}(\varrho_{j})\right|,\\ F(e_{i})|\mu_{H_{\mathbb{P}}}(\varrho_{j})-\mu_{G_{\mathbb{Q}}}(\varrho_{j})|,F(e_{i})|\lambda_{H_{\mathbb{P}}}(\varrho_{j})-\lambda_{G_{\mathbb{Q}}}(\varrho_{j})|. \end{array}\right)\leq 6$$

$\Rightarrow 0\leq d_{1}^{H}\left(H_{P},G_{Q}\right)\leq 1$

**2)** Let $d_{1}^{H}\left(H_{P},G_{Q}\right)=0$, For two CIFHSs $H_{P}$ and $G_{Q}$,

$$\Rightarrow \frac{1}{6m}\sum_{i=1}^{m}\sum_{j=1}^{n}max\left(\begin{array}{l} F(e_{i})\left|\mu_{H_{\mathbb{P}}}^{L}(\varrho_{j})-\mu_{G_{\mathbb{Q}}}^{L}(\varrho_{j})\right|,F(e_{i})\left|\mu_{H_{\mathbb{P}}}^{U}(\varrho_{j})-\mu_{G_{\mathbb{Q}}}^{U}(\varrho_{j})\right|,\\ F(e_{i})\left|\lambda_{H_{\mathbb{P}}}^{L}(\varrho_{j})-\lambda_{G_{\mathbb{Q}}}^{L}(\varrho_{j})\right|,F(e_{i})\left|\lambda_{H_{\mathbb{P}}}^{U}(\varrho_{j})-\lambda_{G_{\mathbb{R}}}^{U}(\varrho_{j})\right|,\\ F(e_{i})|\mu_{H_{\mathbb{P}}}(\varrho_{j})-\mu_{G_{\mathbb{Q}}}(\varrho_{j})|,F(e_{i})|\lambda_{H_{\mathbb{P}}}(\varrho_{j})-\lambda_{G_{\mathbb{Q}}}(\varrho_{j})|. \end{array}\right)=0$$

If and only if, For all *j*,

$max|\mu_{H_{P}}^{L}\left(\varrho_{j}\right)-\mu_{G_{Q}}^{L}\left(\varrho_{j}\right)|=0$ , $max|\mu_{H_{P}}^{U}\left(\varrho_{j}\right)-\mu_{G_{Q}}^{U}\left(\varrho_{j}\right)|=0$, $max|\lambda_{H_{P}}^{L}\left(\varrho_{j}\right)-\lambda_{G_{Q}}^{L}\left(\varrho_{j}\right)|=0$ and $max|\lambda_{H_{P}}^{U}\left(\varrho_{j}\right)-\lambda_{G_{Q}}^{U}\left(\varrho_{j}\right)|=0$, $max|\mu_{H_{P}}\left(\varrho_{j}\right)-\mu_{G_{Q}}\left(\varrho_{j}\right)|=0$, $max|\lambda_{H_{P}}\left(\varrho_{j}\right)-\lambda_{G_{Q}}\left(\varrho_{j}\right)|=0$

which is equivalent to

$max(\mu_{H_{P}}^{L}\left(\varrho_{j}\right))=max(\mu_{G_{Q}}^{L}\left(\varrho_{j}\right))$ , $max(\mu_{H_{P}}^{U}\left(\varrho_{j}\right))=max(\mu_{G_{Q}}^{U}\left(\varrho_{j}\right))$, $max(\lambda_{H_{P}}^{L}\left(\varrho_{j}\right))=max(\lambda_{G_{Q}}^{L}\left(\varrho_{j}\right))$, $max(\lambda_{H_{P}}^{U}\left(\varrho_{j}\right))=max(\lambda_{G_{Q}}^{U}\left(\varrho_{j}\right))$, $max(\mu_{H_{P}}\left(\varrho_{j}\right))=max(\mu_{G_{Q}}\left(\varrho_{j}\right))$, $max(\lambda_{H_{P}}\left(\varrho_{j}\right))=max(\lambda_{G_{Q}}\left(\varrho_{j}\right))$

**3)** For two CIFHSs $H_{P}$ and $G_{Q}$,

$$d_{1}^{H}(H_{P},G_{Q})=\frac{1}{6m}\sum_{i=1}^{m}\sum_{j=1}^{n}max\left(\begin{array}{l} F(e_{i})\left|\mu_{H_{\mathbb{P}}}^{L}(\varrho_{j})-\mu_{G_{\mathbb{Q}}}^{L}(\varrho_{j})\right|,F(e_{i})\left|\mu_{H_{\mathbb{P}}}^{U}(\varrho_{j})-\mu_{G_{\mathbb{Q}}}^{U}(\varrho_{j})\right|,\\ F(e_{i})\left|\lambda_{H_{\mathbb{P}}}^{L}(\varrho_{j})-\lambda_{G_{\mathbb{Q}}}^{L}(\varrho_{j})\right|,F(e_{i})\left|\lambda_{H_{\mathbb{P}}}^{U}(\varrho_{j})-\lambda_{G_{\mathbb{R}}}^{U}(\varrho_{j})\right|,\\ F(e_{i})|\mu_{H_{\mathbb{P}}}(\varrho_{j})-\mu_{G_{\mathbb{Q}}}(\varrho_{j})|,F(e_{i})|\lambda_{H_{\mathbb{P}}}(\varrho_{j})-\lambda_{G_{\mathbb{Q}}}(\varrho_{j})|. \end{array}\right)$$

$$=\frac{1}{6m}\sum_{i=1}^{m}\sum_{j=1}^{n}max\left(\begin{array}{l} F(e_{i})\left|\mu_{G_{\mathbb{P}}}^{L}(\varrho_{j})-\mu_{H_{\mathbb{Q}}}^{L}(\varrho_{j})\right|,F(e_{i})\left|\mu_{G_{\mathbb{P}}}^{U}(\varrho_{j})-\mu_{H_{\mathbb{Q}}}^{U}(\varrho_{j})\right|,\\ F(e_{i})\left|\lambda_{G_{\mathbb{P}}}^{L}(\varrho_{j})-\lambda_{H_{\mathbb{Q}}}^{L}(\varrho_{j})\right|,F(e_{i})\left|\lambda_{G_{\mathbb{P}}}^{U}(\varrho_{j})-\lambda_{H_{\mathbb{R}}}^{U}(\varrho_{j})\right|,\\ F(e_{i})|\mu_{G_{\mathbb{P}}}(\varrho_{j})-\mu_{H_{\mathbb{Q}}}(\varrho_{j})|,F(e_{i})|\lambda_{G_{\mathbb{P}}}(\varrho_{j})-\lambda_{H_{\mathbb{Q}}}(\varrho_{j})|. \end{array}\right)$$

$$\begin{array}{c} =d_{1}^{H}\left(G_{Q},H_{P}\right) \end{array}$$

Hence, $d_{1}^{H}\left(H_{P},G_{Q}\right)=d_{1}^{H}\left(G_{Q},H_{P}\right)$

**4)** $H_{P}\subseteq G_{Q}\subseteq I_{R}$ , then

$max\left[\mu_{H_{P}}^{L}\left(\varrho_{j}\right),\mu_{H_{P}}^{U}\left(\varrho_{j}\right)\right]\subseteq max[\mu_{G_{Q}}^{L}\left(\varrho_{j}\right),max\left(\mu_{G_{Q}}^{U}\left(\varrho_{j}\right)\right]\subseteq max[\mu_{I_{R}}^{L}\left(\varrho_{j}\right),max\left(\mu_{I_{R}}^{U}\left(\varrho_{j}\right)\right]$

$max\left[\lambda_{H_{P}}^{L}\left(\varrho_{j}\right),\lambda_{H_{P}}^{U}\left(\varrho_{j}\right)\right]⊇max[\lambda_{G_{Q}}^{L}\left(\varrho_{j}\right),max\left(\lambda_{G_{Q}}^{U}\left(\varrho_{j}\right)\right]⊇max[\lambda_{I_{R}}^{L}\left(\varrho_{j}\right),max\left(\lambda_{I_{R}}^{U}\left(\varrho_{j}\right)\right]$

Also $max\left(\mu_{H_{P}}\left(\varrho_{j}\right)\right)\geq max\left(\mu_{G_{Q}}\left(\varrho_{j}\right)\right)\geq max\left(\mu_{I_{R}}\left(\varrho_{j}\right)\right)$, and

$max\left(\lambda_{H_{P}}\left(\varrho_{j}\right)\right)\leq max\left(\lambda_{G_{Q}}\left(\varrho_{j}\right)\right)\leq max\left(\lambda_{I_{R}}\left(\varrho_{j}\right)\right)$

Therefore,

$max|\mu_{H_{P}}^{L}\left(\varrho_{j}\right),\mu_{G_{P}}^{L}\left(\varrho_{j}\right)|\leq max|\mu_{H_{Q}}^{L}\left(\varrho_{j}\right),max(\mu_{I_{R}}^{L}\left(\varrho_{j}\right)|,max|\mu_{H_{Q}}^{U}\left(\varrho_{j}\right),max(\mu_{G_{Q}}^{U}\left(\varrho_{j}\right)|\leq$

$max|\mu_{H_{Q}}^{U}\left(\varrho_{j}\right),max(\mu_{I_{R}}^{U}\left(\varrho_{j}\right)|$

$max|\lambda_{H_{P}}^{L}\left(\varrho_{j}\right),\lambda_{G_{P}}^{L}\left(\varrho_{j}\right)|\leq max|\lambda_{H_{Q}}^{L}\left(\varrho_{j}\right),max(\lambda_{I_{R}}^{L}\left(\varrho_{j}\right)|,max|\lambda_{H_{Q}}^{U}\left(\varrho_{j}\right),max(\lambda_{G_{Q}}^{U}\left(\varrho_{j}\right)|\leq$

$max|\lambda_{H_{Q}}^{U}\left(\varrho_{j}\right),max(\lambda_{I_{Q}}^{U}\left(\varrho_{j}\right)|$

$max|\mu_{H_{P}}\left(\varrho_{j}\right),\mu_{G_{Q}}\left(\varrho_{j}\right)|\leq max|\mu_{H_{Q}}\left(\varrho_{j}\right),\mu_{I_{R}}\left(\varrho_{j}\right)|,max|\lambda_{H_{P}}\left(\varrho_{j}\right),\lambda_{G_{Q}}\left(\varrho_{j}\right)|\leq max|\lambda_{H_{Q}}\left(\varrho_{j}\right),\lambda_{I_{R}}\left(\varrho_{j}\right)|$ , Thus,

$$d_{1}^{H}(H_{P},I_{Q})=\frac{1}{6m}\sum_{i=1}^{m}\sum_{j=1}^{n}max\left(\begin{array}{l} F(e_{i})\left|\mu_{H_{P}}^{L}(\varrho_{j})-\mu_{I_{{}_{R}}}^{L}(\varrho_{j})\right|,F(e_{i})\left|\mu_{H_{P}}^{U}(\varrho_{j})-\mu_{I_{R}}^{U}(\varrho_{j})\right|,\\ F(e_{i})\left|\lambda_{H_{P}}^{L}(\varrho_{j})-\lambda_{H_{{}_{R}}}^{L}(\varrho_{j})\right|,F(e_{i})\left|\lambda_{H_{P}}^{U}(\varrho_{j})-\lambda_{I_{R}}^{U}(\varrho_{j})\right|,\\ F(e_{i})|\mu_{H_{P}}(\varrho_{j})-\mu_{I_{{}_{R}}}(\varrho_{j})|,F(e_{i})|\lambda_{H_{P}}(\varrho_{j})-\lambda_{I_{{}_{R}}}(\varrho_{j})|. \end{array}\right)$$

$$\geq \frac{1}{6m}\sum_{i=1}^{m}\sum_{j=1}^{n}max\left(\begin{array}{l} F(e_{i})\left|\mu_{H_{P}}^{L}(\varrho_{j})-\mu_{G_{{}_{Q}}}^{L}(\varrho_{j})\right|,F(e_{i})\left|\mu_{H_{P}}^{U}(\varrho_{j})-\mu_{G_{Q}}^{U}(\varrho_{j})\right|,\\ F(e_{i})\left|\lambda_{H_{P}}^{L}(\varrho_{j})-\lambda_{G_{Q}}^{L}(\varrho_{j})\right|,F(e_{i})\left|\lambda_{H_{P}}^{U}(\varrho_{j})-\lambda_{G_{Q}}^{U}(\varrho_{j})\right|,\\ F(e_{i})|\mu_{H_{P}}(\varrho_{j})-\mu_{G_{Q}}(\varrho_{j})|,F(e_{i})|\lambda_{H_{P}}(\varrho_{j})-\lambda_{G_{Q}}(\varrho_{j})|. \end{array}\right)$$

$$\begin{array}{c} =d_{1}^{H}\left(H_{P},G_{Q}\right) \end{array}$$

Similarly, $d_{1}^{H}\left(H_{P},I_{R}\right)\geq d_{1}^{H}\left(G_{Q},I_{R}\right)$.

Hence, $d_{1}^{H}$ is a valid distance measure.

#### 4.2.2 Hausdorff normalized hamming distance measure

The hausdorff normalized hamming distance can be computed using the expression below:

$$\begin{array}{c} d_{2}^{H}\left(H_{P},G_{Q}\right)=\frac{1}{6mn}\sum_{i=1}^{m}\sum_{j=1}^{n}(\begin{array}{c} max(\begin{array}{c} F\left(e_{i}\right)|\mu_{H_{P}}^{L}\left(\varrho_{j}\right)-\mu_{G_{Q}}^{L}\left(\varrho_{j}\right)|,F\left(e_{i}\right)\left|\mu_{H_{P}}^{U}\left(\varrho_{j}\right)-\mu_{G_{Q}}^{U}\left(\varrho_{j}\right)\right|,\\ F\left(e_{i}\right)|\lambda_{H_{P}}^{L}\left(\varrho_{j}\right)-\lambda_{G_{Q}}^{L}\left(\varrho_{j}\right)|,F\left(e_{i}\right)\left|\lambda_{H_{P}}^{U}\left(\varrho_{j}\right)-\lambda_{G_{Q}}^{U}\left(\varrho_{j}\right)\right|,\\ F\left(e_{i}\right)|\mu_{H_{P}}\left(\varrho_{j}\right)-\mu_{G_{Q}}\left(\varrho_{j}\right)|,F\left(e_{i}\right)\left|\lambda_{H_{P}}\left(\varrho_{j}\right)-\lambda_{G_{Q}}\left(\varrho_{j}\right)\right| \end{array}) \end{array}) \end{array}$$

Here *ϱ* are the elements of ℧ which is a universal set, “n” represents the number of elements of universal sets and “m” represents the number of attributes.

#### 4.2.3 Hausdorff weighted hamming distance measure

The hausdorff weighted hamming distance can be calculated using the expression:

$$\begin{array}{c} d_{3}^{H}\left(H_{P},G_{Q}\right)=\frac{1}{6m}\sum_{i=1}^{m}\sum_{j=1}^{n}C_{i}(\begin{array}{c} max(\begin{array}{c} F\left(e_{i}\right)|\mu_{H_{P}}^{L}\left(\varrho_{j}\right)-\mu_{G_{Q}}^{L}\left(\varrho_{j}\right)|,F\left(e_{i}\right)\left|\mu_{H_{P}}^{U}\left(\varrho_{j}\right)-\mu_{G_{Q}}^{U}\left(\varrho_{j}\right)\right|,\\ F\left(e_{i}\right)|\lambda_{H_{P}}^{L}\left(\varrho_{j}\right)-\lambda_{G_{Q}}^{L}\left(\varrho_{j}\right)|,F\left(e_{i}\right)\left|\lambda_{H_{P}}^{U}\left(\varrho_{j}\right)-\lambda_{G_{Q}}^{U}\left(\varrho_{j}\right)\right|,\\ F\left(e_{i}\right)|\mu_{H_{P}}\left(\varrho_{j}\right)-\mu_{G_{Q}}\left(\varrho_{j}\right)|,F\left(e_{i}\right)\left|\lambda_{H_{P}}\left(\varrho_{j}\right)-\lambda_{G_{Q}}\left(\varrho_{j}\right)\right| \end{array}) \end{array}) \end{array}$$

Here *ϱ* are the elements of ℧ which is a universal set, “n” represents the number of elements of universal sets and “m” represents the number of attributes.

## 5 Development of distance measures for quality control metrics for industrial product manufacture

Metrics for quality control are crucial in product manufacturing to guarantee that the end product satisfies the required criteria for quality. These metrics are measurements and indications that are used to judge a product’s quality as it is being produced. Manufacturers can spot any flaws or problems in their products early on in the production process by using quality control metrics, which enables them to take remedial action and stop the creation of faulty products. As a result, waste is minimized, production costs are decreased, and customer satisfaction is increased. In order to improve their production processes, producers can make modifications that will raise the caliber of their products with the use of quality control metrics. Improved equipment maintenance, faster production lines, and staff training on quality control best practices are a few examples of how to achieve this. Implementing quality control metrics can assist manufacturers in meeting industry standards and regulatory requirements in addition to enhancing product quality. Manufacturers are required to abide by tight quality control rules in many industries; failure to do so may result in penalties, legal action, and harm to the company’s reputation.

Fuzzy distance measures are mathematical tools used in the manufacturing industry for various purposes. They determine the degree of similarity or dissimilarity between two or more entities or objects. In quality control, fuzzy distance measures can identify any quality issues by comparing the similarity of a product’s quality characteristics to its specifications. For production planning, fuzzy distance measures can cluster similar products and identify patterns in production data to improve efficiency and reduce waste. In decision-making, fuzzy distance measures can be used to evaluate the similarity between different products, manufacturing processes, or suppliers to make informed decisions. Fuzzy distance measures can also be used in product design to evaluate the degree of similarity between different design concepts or prototypes. Lastly, in supply chain management, fuzzy distance measures can be used to evaluate the similarity between suppliers or partners to optimize supply chain processes.

The proposed methodology involves constructing a reference set that embodies the ideal qualities of key parameters for cement storage. This reference set is a benchmark against which various cement storage options are compared. To access the quality of the final processed product, suppose that there are “s” batches/alternatives produced, indicated by $\overset{˘}{X_{1}},\overset{˘}{X_{2}},\overset{˘}{X_{3}},\ldots ,\overset{˘}{X_{s}},$ which are assessed by an expert using a set of “t” criteria, denoted by $\overset{˘}{Y_{1}},\overset{˘}{Y_{2}},\overset{˘}{Y_{3}},\ldots ,\overset{˘}{Y_{t}},$. The quality control experts based on their human intuition express their preferences in the form of CIFHS. The preferences are expressed based on the expert’s human intuition where the membership interval is used to express the range in which the membership function should be contained for the quality assessment to be satisfactory while the same non-membership interval and the membership degree express the preferences on contrary to the membership functions. The preferences in the form of CIFHSs are illustrated as:

$$\begin{array}{c} \beta_{st}=(\left\langle \left[\mu_{mn}^{L},\mu_{mn}^{U}\right],\left[\lambda_{mn}^{L},\lambda_{mn}^{U}\right]\right\rangle ,\left\langle \mu_{mn}^{L},\lambda_{mn}^{U}\right\rangle ) \end{array}$$ (2)

where m = 1,2,3,…,s; and n = 1,2,3,…,t.

As a result, all possible rating values for each of the alternatives are compiled in terms of CIFHSs as:

$$\begin{array}{c} \overset{˘}{X_{p}}=(\varrho_{n},\left\langle \left[\mu_{mn}^{L}\left(\varrho_{n}\right),\mu_{mn}^{U}\left(\varrho_{n}\right)\right],\left[\lambda_{mn}^{L}\left(\varrho_{n}\right),\lambda_{mn}^{U}\left(\varrho_{n}\right)\right]\right\rangle ,\left\langle \mu_{mn}^{L}\left(\varrho_{n}\right),\lambda_{mn}^{U}\left(\varrho_{n}\right)\right\rangle |\;\varrho_{n}\in ℧) \end{array}$$ (3)

Let $C_{n}$ be the weight of criteria $\overset{˘}{Y_{n}}$. These weights are essential as they determine the relative importance of each of the criteria that are under review by the expert. The weights can either be provided by the experts or be computed by different decision-making algorithms like Analytical Hierarchy Process (AHP) and Best-Worst Method (BWM) as illustrated by research reported in the literature. Let $C_{n}$ be the weight of criteria $\overset{˘}{Y_{n}}$ such that $C_{n}>0$ and $\sum_{q=1}^{n}C_{n}=1.$ Then, utilising the recommended measures, the following actions are provided to tackle the DM problems:

**Step 1**: Collect all information in terms of CIFHSs relating to each of the alternatives, and therefore an overall decision matrix M is written as:
  $$\begin{array}{c} M=(\begin{array}{cccc} x_{11} & x_{12} & \ldots & x_{1t}\\ x_{21} & x_{22} & \ldots & x_{2t}\\ \vdots & \vdots & \ddots & \vdots \\ x_{s1} & x_{s2} & \ldots & x_{st} \end{array}) \end{array}$$
**Step 2**: Utilizing the proposed distance measures for each of the alternatives against the reference set.
**Step 3**: If the distance between the alternatives is decreasing, it means that the possibilities are nearer to the relevant of the group. As a result, order the possibilities in decreasing distance order.

### 5.1 Quality control for storage of manufactured cement

The quality control of cement manufacture is a critical aspect of the cement industry, as it affects the strength, durability, and overall performance of the final product. Several factors can influence the quality control of cement manufacturing [54].

1. **Raw materials**: The quality of raw materials used in cement manufacturing plays a crucial role in determining the quality of the finished product. The chemical composition, fineness, and consistency of raw materials such as limestone, clay, and gypsum must be carefully monitored to ensure they meet the required standards.
2. **Production process**: The production process used to manufacture cement can also impact the quality of the final product. Factors such as temperature, mixing time, and grinding process can all influence the quality of the cement.
3. **Quality control procedures**: Cement manufacturers must have robust quality control procedures to ensure that the finished product meets the required standards. These procedures should include regularly testing raw materials and finished products to meet the required specifications.
4. **Environmental factors**: Environmental factors such as humidity, temperature, and air quality can also affect cement quality. These factors can impact the setting time, strength development, and consistency of the cement.
5. **Storage and transportation**: The storage and transportation of cement can also impact its quality. Cement should be stored in a dry, cool, and well-ventilated area to prevent moisture from affecting its quality. Additionally, it should be transported in clean, dry, and well-maintained trucks to prevent contamination.

Now, cement storage is a critical issue as the finished product needs to be handled carefully and safely shipped to consumers without any damage. A number of variables are considered when storing cement, and its essential to keep a strict quality control check over them as it greatly influences the final performance when used by the consumer. To compute the quality control metrics for storage, consider *ϱ*_1_, *ϱ*_2_, *ϱ*_3_ be the cement batches produced by an industry. Let P be the set of parameters given by P = {*P*_1_ (Temperature), *P*_2_ (Humidity), *P*_3_ (Air Circulation in the storage site), (Enclosure Type)} and their sub-parametric sets as

*P*_1_ = {(10–30%), (30–50%),(greater than 50%)}, *P*_2_ = {Temperature, Pressure, Humidity, Greenhouse Emissions} and *P*_3_ = {Humidity, Air Circulation in the warehouse, Temperature, Enclosure Types} which are represented in the form of CIFHSS as $H_{P},I_{P}$ and $J_{P}$ are optimal settings reported in literature for storage of cement.

For the sake of brevity, four tuples of attributes of CIFHS are computed as follows:

$$\begin{array}{c} H_{P}=\{\begin{array}{c} H_{{\hat{p}}_{1}}=\left\{\left\langle \varrho_{1},\left(\left[0.3,0.5\right],\left[0.1,0.5\right]\right),\left(0.4,0.6\right)\right\rangle ,\left\langle \varrho_{2},\left(\left[0.3,0.4\right],\left[0.1,0.5\right]\right),\left(0.4,0.5\right)\right\rangle ,\left\langle \varrho_{3},\left(\left[0.2,0.6\right],\left[0.1,0.4\right]\right),\left(0.3,0.6\right)\right)\right\rangle \},\\ H_{{\hat{p}}_{2}}=\left\{\left\langle \varrho_{1},\left(\left[0.1,0.4\right],\left[0.4,0.6\right]\right),\left(0.2,0.7\right)\right\rangle ,\left\langle \varrho_{2},\left(\left[0.2,0.4\right],\left[0.5,0.6\right]\right),\left(0.1,0.4\right)\right\rangle ,\left\langle \varrho_{3},\left(\left[0.3,0.8\right],\left[0.1,0.2\right]\right),\left(0.6,0.1\right)\right\rangle \right\},\\ H_{{\hat{p}}_{3}}=\left\{\left\langle \varrho_{1},\left(\left[0.2,0.6\right],\left[0.1,0.3\right]\right),\left(0.2,0.6\right)\right\rangle ,\left\langle \varrho_{2},\left(\left[0.2,0.4\right],\left[0.1,0.4\right]\right),\left(0.3,0.5\right)\right\rangle ,\left\langle \varrho_{3},\left(\left[0.1,0.2\right],\left[0.3,0.8\right]\right),\left(0.3,0.5\right)\right\rangle \right\},\\ H_{{\hat{p}}_{4}}=\left\{\left\langle \varrho_{1},\left(\left[0.1,0.4\right],\left[0.3,0.6\right]\right),\left(0.1,0.8\right)\right\rangle ,\left\langle \varrho_{2},\left(\left[0.1,0.5\right],\left[0.1,0.3\right]\right),\left(0.3,0.5\right)\right\rangle ,\left\langle \varrho_{3},\left(\left[0.2,0.3\right],\left[0.2,0.7\right]\right),\left(0.2,0.4\right)\right\rangle \right\} \end{array}\} \end{array}$$

$$\begin{array}{c} I_{P}=\{\begin{array}{c} H_{{\hat{p}}_{1}}=\left\{\left\langle \varrho_{1},\left(\left[0.2,0.7\right],\left[0.1,0.2\right]\right),\left(0.3,0.5\right)\right\rangle ,\left\langle \varrho_{2},\left(\left[0.3,0.6\right],\left[0.2,0.4\right]\right),\left(0.4,0.6\right)\right\rangle ,\left\langle \varrho_{3},\left(\left[0.4,0.5\right],\left[0.2,0.4\right]\right),\left(0.6,0.3\right)\right)\right\rangle \},\\ H_{{\hat{p}}_{2}}=\left\{\left\langle \varrho_{1},\left(\left[0.1,0.5\right],\left[0.3,0.5\right]\right),\left(0.5,0.4\right)\right\rangle ,\left\langle \varrho_{2},\left(\left[0.2,0.4\right],\left[0.5,0.6\right]\right),\left(0.1,0.9\right)\right\rangle ,\left\langle \varrho_{3},\left(\left[0.2,0.6\right],\left[0.1,0.3\right]\right),\left(0.5,0.4\right)\right\rangle \right\},\\ H_{{\hat{p}}_{3}}=\left\{\left\langle \varrho_{1},\left(\left[0.6,0.8\right],\left[0.1,0.2\right]\right),\left(0.4,0.5\right)\right\rangle ,\left\langle \varrho_{2},\left(\left[0.1,0.6\right],\left[0.1,0.3\right]\right),\left(0.2,0.8\right)\right\rangle ,\left\langle \varrho_{3},\left(\left[0.1,0.3\right],\left[0.2,0.7\right]\right),\left(0.2,0.4\right)\right\rangle \right\},\\ H_{{\hat{p}}_{4}}=\left\{\left\langle \varrho_{1},\left(\left[0.3,0.4\right],\left[0.5,0.6\right]\right),\left(0.6,0.3\right)\right\rangle ,\left\langle \varrho_{2},\left(\left[0.7,0.8\right],\left[0.1,0.2\right]\right),\left(0.7,0.2\right)\right\rangle ,\left\langle \varrho_{3},\left(\left[0.6,0.7\right],\left[0.2,0.3\right]\right),\left(0.1,0.5\right)\right\rangle \right\} \end{array}\} \end{array}$$

$$\begin{array}{c} J_{P}=\{\begin{array}{c} H_{{\hat{p}}_{1}}=\left\{\left\langle \varrho_{1},\left(\left[0.2,0.5\right],\left[0.1,0.4\right]\right),\left(0.6,0.2\right)\right\rangle ,\left\langle \varrho_{2},\left(\left[0.1,0.3\right],\left[0.2,0.7\right]\right),\left(0.7,0.1\right)\right\rangle ,\left\langle \varrho_{3},\left(\left[0.5,0.8\right],\left[0.1,0.2\right]\right),\left(0.9,0.1\right)\right)\right\rangle \},\\ H_{{\hat{p}}_{2}}=\left\{\left\langle \varrho_{1},\left(\left[0.4,0.5\right],\left[0.1,0.3\right]\right),\left(0.6,0.3\right)\right\rangle ,\left\langle \varrho_{2},\left(\left[0.3,0.6\right],\left[0.2,0.4\right]\right),\left(0.2,0.7\right)\right\rangle ,\left\langle \varrho_{3},\left(\left[0.4,0.6\right],\left[0.2,0.3\right]\right),\left(0.2,0.8\right)\right\rangle \right\},\\ H_{{\hat{p}}_{3}}=\left\{\left\langle \varrho_{1},\left(\left[0.3,0.8\right],\left[0.1,0.2\right]\right),\left(0.3,0.6\right)\right\rangle ,\left\langle \varrho_{2},\left(\left[0.1,0.5\right],\left[0.3,0.5\right]\right),\left(0.3,0.4\right)\right\rangle ,\left\langle \varrho_{3},\left(\left[0.2,0.4\right],\left[0.1,0.3\right]\right),\left(0.4,0.3\right)\right\rangle \right\},\\ H_{{\hat{p}}_{4}}=\left\{\left\langle \varrho_{1},\left(\left[0.3,0.6\right],\left[0.1,0.3\right]\right),\left(0.1,0.5\right)\right\rangle ,\left\langle \varrho_{2},\left(\left[0.4,0.5\right],\left[0.2,0.4\right]\right),\left(0.7,0.2\right)\right\rangle ,\left\langle \varrho_{3},\left(\left[0.7,0.8\right],\left[0.1,0.2\right]\right),\left(0.8,0.1\right)\right\rangle \right\} \end{array}\} \end{array}$$

Now, a reference set is used for comparison of the alternatives so the closest to the ideal alternative is selected. Assume the properties of an ideal storage facility for cement are recorded in the form of an n-tuple to see the highest level of n-tuple compliance with $H_{P}$, $I_{P}$ and $J_{P}$. For this purpose, a list is compiled with different storage options they have currently available out of which their aim is to the select the closest to the ideal option. Most of the data for considering the similarity between the presented options is available in the form of human intuition allowing room for uncertainty which is best dealt using fuzzy structures. The list in the form of CIFHSS is shown below:

$$\begin{array}{c} R_{P}=\{\begin{array}{c} H_{{\hat{p}}_{1}}=\left\{\left\langle \varrho_{1},\left(\left[0.1,0.3\right],\left[0.2,0.5\right]\right),\left(0.6,0.3\right)\right\rangle ,\left\langle \varrho_{2},\left(\left[0.5,0.6\right],\left[0.3,0.4\right]\right),\left(0.2,0.7\right)\right\rangle ,\left\langle \varrho_{3},\left(\left[0.3,0.8\right],\left[0.1,0.2\right]\right),\left(0.2,0.7\right)\right)\right\rangle \},\\ H_{{\hat{p}}_{2}}=\left\{\left\langle \varrho_{1},\left(\left[0.3,0.6\right],\left[0.2,0.3\right]\right),\left(0.8,0.1\right)\right\rangle ,\left\langle \varrho_{2},\left(\left[0.1,0.4\right],\left[0.2,0.6\right]\right),\left(0.3,0.5\right)\right\rangle ,\left\langle M\varrho_{3},\left(\left[0.4,0.5\right],\left[0.3,0.4\right]\right),\left(0.3,0.6\right)\right\rangle \right\},\\ H_{{\hat{p}}_{3}}=\left\{\left\langle \varrho_{1},\left(\left[0.1,0.6\right],\left[0.2,0.3\right]\right),\left(0.3,0.5\right)\right\rangle ,\left\langle \varrho_{2},\left(\left[0.2,0.5\right],\left[0.3,0.5\right]\right),\left(0.1,0.4\right)\right\rangle ,\left\langle \varrho_{3},\left(\left[0.1,0.8\right],\left[0.1,0.2\right]\right),\left(0.2,0.7\right)\right\rangle \right\},\\ H_{{\hat{p}}_{4}}=\left\{\left\langle \varrho_{1},\left(\left[0.4,0.5\right],\left[0.2,0.4\right]\right),\left(0.1,0.8\right)\right\rangle ,\left\langle \varrho_{2},\left(\left[0.6,0.7\right],\left[0.1,0.3\right]\right),\left(0.7,0.1\right)\right\rangle ,\left\langle \varrho_{3},\left(\left[0.5,0.8\right],\left[0.1,0.2\right]\right),\left(0.4,0.6\right)\right\rangle \right\} \end{array}\} \end{array}$$

Now, as explained above, the purpose of this computation is to figure out which of the enclosures out of a set of given options $H_{P}$, $I_{P}$ and $J_{P}$ would perform best for storage of cement products compared to the ones ideally reported in literature. The proposed non-weighted measurements are used to calculate $H_{P}$, $I_{P}$ and $J_{P}$ from ideal enclosure properties. Table 1 presents the summarized results of the non-weighed distance measures indicating $J_{P}$ is the best performer compared to the other set of alternatives ($H_{P}$, $I_{P}$). Suppose weights are assigned as 0.1, 0.4, and 0.5 to the criteria under consideration. In that case, the results may vary as illustrated by values of *d*_6_, $d_{5}^{H}$, $d_{6}^{H}$ whereby inclusion of a significant weight of a criteria leads to different results when compared to the ideal scenario of storage properties of cement.

**Table 1:** Calculated distance measures for cement storage enclosure quality control.

Distance Measures		Measurement Values of R from		Ranking
	*H*_*P*_	*I*_*P*_	*J*_*P*_	
*d*_1_	0.5875	0.55	0.470833	*J*_*P*_<*I*_*P*_<*H*_*P*_
*d*_2_	0.195833	0.183333	0.156944	*J*_*P*_<*I*_*P*_<*H*_*P*_
*d*_3_	0.206667	0.173333	0.172917	*J*_*P*_<*I*_*P*_<*H*_*P*_
$d_{1}^{H}$	0.179167	0.175	0.1625	*J*_*P*_<*I*_*P*_<*H*_*P*_
$d_{2}^{H}$	0.059722	0.058333	0.054167	*J*_*P*_<*I*_*P*_<*H*_*P*_
$d_{3}^{H}$	0.062917	0.054583	0.06125	*I*_*P*_<*J*_*P*_<*H*_*P*_

By employing the hybrid cubic intuitionistic fuzzy hypersoft set structure, the study accommodates and captures the inherent uncertainties and complexities associated with quality control in practical applications, making it highly suitable for real-world industrial environments. This approach offers a powerful tool to assess the similarity between each cement storage site and the reference set of parameters. The degree of similarity determined through this process is crucial in determining whether a particular product should be accepted or rejected in the industrial production line. Products exhibiting a higher degree of similarity to the reference set are more likely to meet the desired quality standards and, thus, be accepted for further processing or distribution. Decision-makers can evaluate the similarities and differences between various cement storage options and the reference set in detail by incorporating distance measures into the hybrid cubic intuitionistic fuzzy hypersoft set structure. This allows for the optimization of quality control procedures. As a result, this study advances quality control methodologies and has a great deal of potential to improve the overall efficacy and efficiency of industrial production lines, which will ultimately result in greater product quality and customer satisfaction. The hybrid CIFHS structure’s incorporation of distance measures enables a detailed assessment of the parallels and divergences between cement storage choices and the reference set, giving decision-makers insightful information for improving quality control procedures. As a result, this study contributes to the advancement of quality control techniques. It holds significant promise for enhancing the overall efficiency and effectiveness of industrial production lines, ultimately leading to higher product quality and customer satisfaction.

While the developed structure holds great promise as highlighted in the introduction, it does have limitations. First, due to the combination of multiple mathematical structures, CIFHSS might introduce increased complexity in the mathematical framework. CIFHSS might introduce increased complexity in the mathematical framework. Secondly, this framework is mathematically limited to work if the sub-parameters are disjoint, requiring significant tuning of parameters before using the framework. Also, as with any fuzzy-based approach, the computational burden increases with larger datasets and more complex systems. While the study highlights the versatility of CIFHSS in quality control management, its generalization to diverse industrial domains might require further investigation.

Compared to the structures reported in the literature, CIFHSS stands out as a highly versatile and powerful framework for handling uncertainty and vagueness in complex data scenarios. By seamlessly integrating the key characteristics of several hybrid fuzzy structures, including cubic sets, hypersoft sets, and intuitionistic fuzzy sets, CIFHSS offers a unique and sophisticated approach that enhances the design and incorporation of human intuitionistic data in decision-making processes. These multiple hybrid fuzzy paradigms are combined inside CIFHSS to produce a complex mathematical framework that more fully reflects uncertainty, ambiguity, and imprecision. The use of cubic sets enables more accurate data modeling by allowing for a more detailed depiction of membership degrees using cubic functions. Aspects of soft computing are also introduced through the introduction of hypersoft sets, improving the flexibility with which many qualities and their sub-attributes can be handled. Furthermore, because it takes into account both membership and non-membership information, the CIFHSS’s incorporation of intuitionistic fuzzy sets allows a richer comprehension and analysis of ambiguous data. This thorough representation is essential for capturing the instincts and preferences of the decision-maker, resulting in more precise and well-informed decision-making processes. The application of CIFHSS across a variety of industrial fields, where complex data frequently necessitates multifaceted analysis and decision-making, further demonstrates its adaptability. Its capacity to manage ambiguous, vague, and imprecise data in a more comprehensible format offers effective and efficient solutions for practical issues including pattern detection, risk analysis, and quality control management.

## 6 Conclusion

In this study, we developed a hybrid concept that merges Cubic Intuitionistic Fuzzy Set (CIFS) with Soft Set (SS) to create the novel Cubic Intuitionistic Fuzzy Hypersoft Set. The main goal was to improve the quality control management system for industrial applications by accurately capturing and modeling uncertainty, ambiguity, and imprecision in data. With the advent of CIFHSS, we were able to manage many unique qualities within a cubic set environment at the sub-attribute level, giving complicated industrial systems a more detailed and accurate depiction. The described operations, such as internal, partly internal, external, complement, direct sum, and product, increased CIFHSS’s adaptability and the range of possible real-world scenarios in which it may be used. The numerical simulations demonstrated how useful and effective CIFHSS is for quality control management. The data’s possible flaws, outliers, and variances were effectively found by using the six distance measures described by CIFHSS. The ability to quantify the differences between data points and assess the consistency of production processes empowered us to make informed decisions, optimize manufacturing processes, and adhere to regulatory requirements effectively. The significant findings of this paper lie in the versatility of CIFHSS as an efficient tool for decision-making, risk analysis, and process optimization across a wide range of industrial applications. By effectively handling uncertainty and vagueness in data, CIFHSS enables manufacturers to maintain consistent production, meet customer expectations, ensure regulatory compliance, and safeguard their reputation and profitability.

## Supporting information

S1 File (PDF)
