# Supplementary material for: Development of hamming and hausdorff distance metrics for cubic intuitionistic fuzzy hypersoft set in cement storage quality control: Development and evaluation
Source: PLoS One. 2023 Sep 25;18(9):e0291817. doi: 10.1371/journal.pone.0291817 (PMC10519612; doi:10.1371/journal.pone.0291817)
Supplement: S1 File — (PDF) [file pone.0291817.s001.pdf]

Manuscript Title: Development of Hamming and Hausdorff Distance Metrics for Cubic Intuitionistic Fuzzy Hypersoft Set in Cement Storage Quality Control: Development and Evaluation

Authors: Muhammad Haris Saeed, Muhammad Saeed, Misbah Khalid, Ibrahim Mekawy

Research Highlights:

1. Introducing a hybrid Cubic Intuitionistic Fuzzy Set with a Soft Set Structure.
2. Defined aggregation operators, set operations, and distance measures for the CIFHSS.
3. Elaborating versatility of developed CIFHSS structure through applications.
4. Applying 6 different CIFHSS distance metrics for industrial product manufacture quality control.
